# Cell-to-cell transmitted alpha-synuclein recapitulates experimental Parkinson’s disease

**DOI:** 10.1038/s41531-023-00618-6

**Published:** 2024-01-06

**Authors:** Natalia Cecilia Prymaczok, Pablo Nicolas De Francesco, Samanta Mazzetti, Marie Humbert-Claude, Liliane Tenenbaum, Graziella Cappelletti, Eliezer Masliah, Mario Perello, Roland Riek, Juan Atilio Gerez

**Affiliations:** 1 Institute of Molecular Physical Science, Department of Chemistry and Applied Biosciences, ETH Zurich, Zurich, Switzerland; 2 Laboratory of Neurophysiology of the Multidisciplinary Institute of Cell Biology (IMBICE), dependent of the Argentine Research Council (CONICET), Scientific Research Commission and University of La Plata Buenos Aires, La Plata, Argentina; 3 Department of Biosciences, Università degli Studi di Milano, Milano, Italy; 4 Fondazione Grigioni per il Morbo di Parkinson, Milano, Italy; 5 Laboratory of Neurotherapies and NeuroModulation, Clinical Neuroscience Department, Center for Neuroscience Research, Lausanne University Hospital, Lausanne, Switzerland; 6 Division of Neurosciences, National Institute on Aging/NIH, 7201 Wisconsin Ave, Bethesda, MD USA

**Keywords:** Parkinson's disease, Genetic vectors

## Abstract

Parkinson’s disease is characterized by a progressive accumulation of alpha-Synuclein (αSyn) neuronal inclusions called Lewy bodies in the nervous system. Lewy bodies can arise from the cell-to-cell propagation of αSyn, which can occur via sequential steps of secretion and uptake. Here, by fusing a removable short signal peptide to the N-terminus of αSyn, we developed a novel mouse model with enhanced αSyn secretion and cell-to-cell transmission. Expression of the secreted αSyn in the mouse brain was under the control of a novel hybrid promoter in combination with adeno-associated virus serotype 9 (AAV9). This combination of promoter and viral vector induced a robust expression in neurons but not in the glia of injected mice. Biochemical characterization of the secreted αSyn revealed that, in cultured cells, this protein is released to the extracellular milieu via conventional secretion. The released αSyn is then internalized and processed by acceptor cells via the endosome–lysosome pathway indicating that the secreted αSyn is cell-to-cell transmitted. The secreted αSyn is aggregation-prone and amyloidogenic, and when expressed in the brain of wild-type non-transgenic mice, it induces a Parkinson’s disease-like phenotype that includes a robust αSyn pathology in the substantia nigra, neuronal loss, neuroinflammation, and motor deficits, all the key features of experimental animal models of Parkinson’s disease. In summary, a novel animal model of Parkinson’s disease based on enhanced cell-to-cell transmission of αSyn was developed. The neuron-produced cell-to-cell transmitted αSyn triggers all phenotypic features of experimental Parkinson’s disease in mice.

## Introduction

Parkinson’s disease, the second most common neurodegenerative disorder in humans, is pathologically characterized by a massive loss of dopaminergic neurons of the substantia nigra pars compacta (SNpc). In Parkinson’s disease neurodegeneration is preceded and accompanied by a progressive accumulation of alpha-synuclein (αSyn) neuronal inclusions termed Lewy bodies (LBs) and Lewy neurites (LNs) in the peripheral and central nervous systems^1–3^. A plethora of clinical and experimental evidence has etiologically linked αSyn, a 14 kDa protein highly expressed in presynaptic terminals, to Parkinson’s disease. This includes that LBs and LNs are invariably found in idiopathic and familial forms of the disease^4^, and certain polymorphisms and autosomal dominant mutations or multiplications of the αSyn-encoding gene lead to increased Parkinson’s disease risk and early onset^5–9^.

Primary works on the neuroanatomical distribution of LB/LNs revealed that Lewy bodies appear in asymptomatic early stages of the disease, where they are found mainly in the peripheral nervous system^3,10–13^. LB/LNs appear to self-propagate and spread towards and through the central nervous system following a topologically and temporally predictable pattern that correlates with disease progression^14^. According to Braak and co-workers, six stages of Parkinson’s disease progression can be defined according to the extent and topographical distribution of the inclusions^14,15^.

Originally considered an intracellular protein, it was recently shown that αSyn is also present in the extracellular milieu and cerebrospinal fluid from both healthy subjects and Parkinson’s disease patients^16–19^. Although a physiological function(s) of the αSyn that is released by the neurons remains enigmatic, extracellular αSyn can initiate LB pathogenesis and spreading^20^, two processes that result from the αSyn cell-to-cell transmission via secretion and uptake^21–25^. The intercellular transmission of αSyn is supported by the acquisition of LB by embryonic mesencephalic neurons grafted into Parkinson’s disease patient’s brains^26–29^. As a consequence of the “host-to-graft” transmission of αSyn, new LB-like inclusions are formed in healthy neuronal cells grafted into the brain of transgenic mice with robust αSyn pathology^30,31^.

Due to its crucial role in LB pathogenesis and Parkinson’s disease progression, several animal models have been developed to study the cell-to-cell transmission of αSyn in the mammalian nervous system. Most of these models are based on the administration of exogenous material as a source of extracellular αSyn. They include the intracerebral injection of brain homogenates and αSyn pre-formed fibrils or PFFs, which are produced from recombinant αSyn monomers produced in bacteria. Though valuable tools, these models preclude investigations on how the extracellular αSyn produced by the host impacts the mammalian brain. To overcome this limitation, we developed a novel mouse model of Parkinson’s disease with enhanced cell-to-cell transmission of αSyn. Importantly, the secreted αSyn is produced by the neurons from the host. The model is based on the ectopic expression of an αSyn variant with enhanced secretion in non-transgenic wild-type mice. Expression of this protein is under the control of a novel promoter that in combination with the adeno-associated virus serotype 9 (AAV9), drives a robust expression in neurons but not in the glia of these animals. In order to enhance its secretion, a removable short signal peptide was fused to the N-terminus of αSyn, and a substantial release of this protein into the extracellular milieu has been achieved. This αSyn variant is released to the extracellular milieu via conventional secretion and then internalized and processed by acceptor cells via the endosome–lysosome pathway. Thus, the αSyn variant that was developed is cell-to-cell transmitted. The secreted αSyn is aggregation-prone and amyloidogenic, and when expressed in the brain of wild-type non-transgenic mice, it induces a Parkinson’s disease-like phenotype that includes a robust αSyn pathology in the substantia nigra, neuronal loss, neuroinflammation, and motor deficits, all are the cardinal features of experimental animal models of Parkinson’s disease. We anticipate that the model will be useful for elucidating the mechanism of Parkinson’s disease progression and αSyn cell-to-cell transmission in vivo and that it will guide the discovery of novel therapeutics for synucleinopaties.

## Results

### sp-tagged αSyn is efficiently secreted and internalized by acceptor cells

**Fig. 1:**
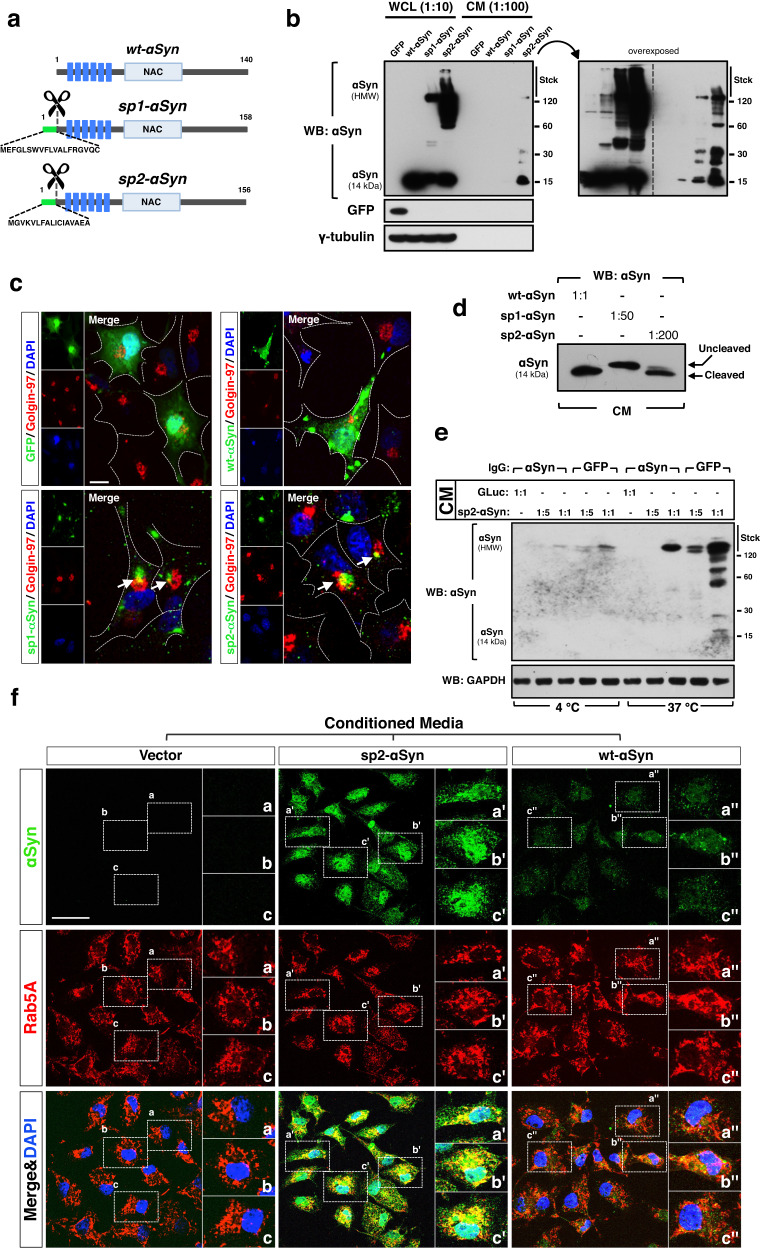
sp-tagged αSyn is secreted and internalized. **a** Schematic representation of the αSyn variants used in this work. Structural elements shown are the signal peptides sp1 and sp2 (green), signal peptide cleavage site (scissors), αSyn KTKEGV repeats motifs (dark blue), and non-amyloid-β component (NAC, light blue). The aminocidic sequences of sp1 and sp2 are shown. **b** Representative Western blot analyses (WB) of whole cell lysate (WCL, 1:10 from the total lysate) and conditioned media (CM, 1:100 from the total CM) of Cos7 cells expressing GFP or wt-, sp1- or sp2-αSyn. Stck, stacking gel. **c** Representative confocal microscopy images of Cos7 cells expressing GFP, wt-, sp1- or sp2-αSyn. In order to visualize only the newly synthetized αSyn and not the one released and subsequently acquired by uptake, the media of the cultures was replaced every 30 min. Arrows indicate co-localization between αSyn and golgin-97. DAPI was used for nuclear staining. Scale bar: 10 μm. **d** WB of CM from Cos7 cells expressing wt-, sp1- or sp2-αSyn. As CM from sp1- or sp2-αSyn-expressing cells contained higher levels of αSyn (**b**), different dilutions (1:50 and 1:200, respectively) of these CM were used in order to load equal amounts of αSyn in all conditions. CM containing wt-αSyn was not diluted (1:1). Only the part of the gel where monomeric αSyn (14 kDa) is observed is shown. **e** Representative WB of WCL from human neuroblastoma SH-SY5Y cells treated with CM collected from GLuc or sp2-αSyn-expressing Cos7 cells. The CM were pre-treated for 4 h with antibodies (indicated as IgG) against αSyn or GFP. After antibody treatment, two amounts of the CM (a dilution 1:5 and a non-diluted sample denoted as 1:1) were used to treat SH-SY5Y cells. Treated cells were grown at 37 or 4 **°**C for 2 h as active uptake is inhibited at low temperatures. Stck, stacking gel. **f** Representative confocal microscopy images of Cos7 cells treated with CM from control cells transfected with empty vector (Vector) or cells expressing sp-2 or wt-αSyn. Double immunofluorescence was carried out with antibodies specific for αSyn (green) and the endosomal small GTPase Rab5A (red). Nuclei were visualized by DAPI staining. Scale bar: 50 μm.

In order to study in vivo potential pathophysiological roles of the αSyn that is cell-to-cell transmitted and produced by the neurons of the host, we developed cellular and animal models by expressing variants of αSyn that are constitutively secreted to the extracellular milieu. This was achieved by fusing a removable short signal peptide (sp) to the N-terminal end of human wild-type αSyn (wt-αSyn). Two sp that have been extensively used in mammalian cells were chosen; (1) signal peptide 1 (sp1), an optimized sp derived from the heavy and light chains of therapeutically optimized immunoglobulins^32^ and (2) signal peptide 2 (sp2), the short secretion sequence of the luciferase of the marine copepod, *Gaussia princeps*^33,34^ (Fig. 1a). In proof-of-principle experiments we transiently transfected expression vectors encoding sp1- and sp2-tagged αSyn in cultured mammalian Cos7 and SH-SY5Y cells. Cos7 are not neuronal cells but, compared to SH-SY5Y cells, a much higher transfection efficiency and expression levels of the ectopically expressed genes can be obtained with these cells. Thus, the behavior of the sp-tagged αSyn can be studied at different expression levels. 48 h later the conditioned media (CM) of the transfected cells were isolated, and the cells were harvested. We found a substantial accumulation of sp1-tagged αSyn (sp1-αSyn) and sp2-tagged αSyn (sp2-αSyn) in the CM of the cells (Fig. 1b lanes 7 and 8, and Supplementary Fig. 1a lanes 6 and 8). This indicated that both signal peptides were able to drive a robust secretion of αSyn into the CM. As it is naturally secreted^21,35–37^, wt-αSyn was also detected in the CM of cells overexpressing this protein, but to a much lesser extent compared to the sp-tagged αSyn (Fig. 1b lane 6 and Supplementary Fig. 1a lane 5). Surprisingly, stable high molecular weight (HMW) species of αSyn were observed in both whole cell lysates (WCL) and CM of sp1-αSyn, sp2-αSyn, and wt-αSyn, expressing cells. Levels of HMW species were, however, remarkably higher in cells expressing the secreted αSyn variants (Fig. 1b lanes 3 and 4, and lanes 7 and 8 and Supplementary Fig. 1a lanes 2 and 4, and 6 and 8). These HMW species might result from two non-mutually exclusive processes named protein aggregation and post-translational modifications, and we studied both of them. Quantification of total levels of the unmodified protein was carried out by parallel reaction monitoring (PRM) mass spectrometry, which showed that in cells the unmodified forms of wt-αSyn are more abundant compared to sp2-αSyn. In CM, on the contrary, sp2-αSyn is more abundant (Supplementary Fig. 1b, c). PRM targeting the wt-αSyn-exclusive N-terminally acetylated tryptic peptide confirmed these results (Supplementary Fig. 1d). Supporting that sp1-αSyn and sp2-αSyn are secreted, both αSyn variants localized in well-defined intra-cytoplasmic structures associated to the Golgi apparatus, as indicated by their co-localization with the Golgi resident protein Golgin-97 (Fig. 1c and Supplementary Fig. 1e). In contrast, the localization of the control protein eGFP and wt-αSyn was diffuse and throughout the cytoplasm and nucleus. A pre-treatment with monensin, a potent inhibitor of the secretory pathway, led to a substantial intracellular accumulation of the secreted αSyn variants (Supplementary Fig. 1f). Surprisingly, higher levels of HMW species of wild type αSyn were also observed in monensin-treated cells (Supplementary Fig. 1f lanes 6 vs. 2 and Supplementary Fig. 1g), suggesting that the secretory pathway might have an effect on αSyn aggregation in normal cells.

Signal peptides are removed from the mature protein along the secretory pathway by specific peptidases, and as expected for sp1 and sp2, both sp were cleaved off upon secretion, although with different efficiencies (Fig. 1d). While removal of sp1 was minimal, sp2 was readily removed (>95%) and therefore the monomers of sp2-αSyn found in CM were indistinguishable from wt-αSyn by SDS–PAGE and Western blot (WB). This was also confirmed by PRM (Supplementary Fig. 1h). Cleavage is attributed to the secretory pathway as the sp2 tag is not cleaved off in a transexpressed^38^ recombinant sp2-αSyn (Supplementary Fig. 1i).

We then asked whether neuronal cells could internalize the αSyn that had been released from the cells, and treated SH-SY5Y human neuroblastoma cells with CM from Cos7 cells expressing sp2-αSyn. Sp1-αSyn was not used in this experiment because the signal peptide was not fully removed from this protein and it could affect uptake. These CM were pre-treated with antibodies against eGFP (control) or αSyn in order to inhibit exogenous αSyn uptake by antibodies and thereby confirm αSyn internalization^39^. We found high levels of αSyn in recipient SH-SY5Y neuronal cells treated with CM containing sp2-αSyn and pre-treated with anti-GFP antibodies (Fig. 1e lanes 9 and 10), while αSyn levels were substantially lower when the CM was pre-absorbed with anti-αSyn antibodies. This confirmed cellular uptake of sp2-αSyn as well as antibody-mediated inhibition of αSyn internalization (Fig. 1e lanes 7 and 8 vs. 9 and 10). Accumulation of αSyn in recipient cells was strongly inhibited at 4 °C (Fig. 1e lanes 2–5 vs. 7–10), in agreement with active mechanisms of uptake. Confocal microscopy analyses of Cos7 cells treated with CM containing sp2-αSyn showed that the internalized αSyn strongly co-localized with the endosomal Rab GTPase Rab5A (Fig. 1f and Supplementary Fig. 1j). This suggested that the secreted sp2-αSyn can be internalized by endocytosis in acceptor cells, as previously shown for extracellular αSyn aggregates^22,24,25^.

### Secreted αSyn is aggregation-prone, amyloidogenic, and cell-to-cell transmitted

We next investigated the cytotoxicity of the secreted αSyn. We first analyzed the contribution of the sp2-tag to αSyn because, despite the fact that it is cleaved off upon secretion, it could affect αSyn aggregation kinetics. Fibrillization curves monitored by the amyloid dye thioflavin-T indicated, to our surprise, that recombinant sp2-αSyn aggregates slower than recombinant wt-αSyn when incubated in PBS buffer (Supplementary Fig. 2a). Nuclear magnetic resonance (NMR) experiments (two dimensional [^15^N,^1^H]-heteronuclear multiple quantum coherence (HMQC) of ^15^N-labeled wt-αSyn and sp2-αSyn also demonstrated that in PBS buffer recombinant sp2-αSyn behaves like wt-αSyn and does not aggregates rapidly (Supplementary Fig. 2b). In vitro NMR experiments were supported by in-cell NMR that showed a similar behavior between ^15^N-labeled recombinant wt-αSyn and sp2-αSyn in mammalian cells (Supplementary Fig. 2c). Thus, we concluded that the sp2-tag does not enhance αSyn aggregation but it is induced by other factors such as those imprinted along the secretory pathway (e.g. glycosylation, fast aggregation due to low pH^40^).

**Fig. 2:**
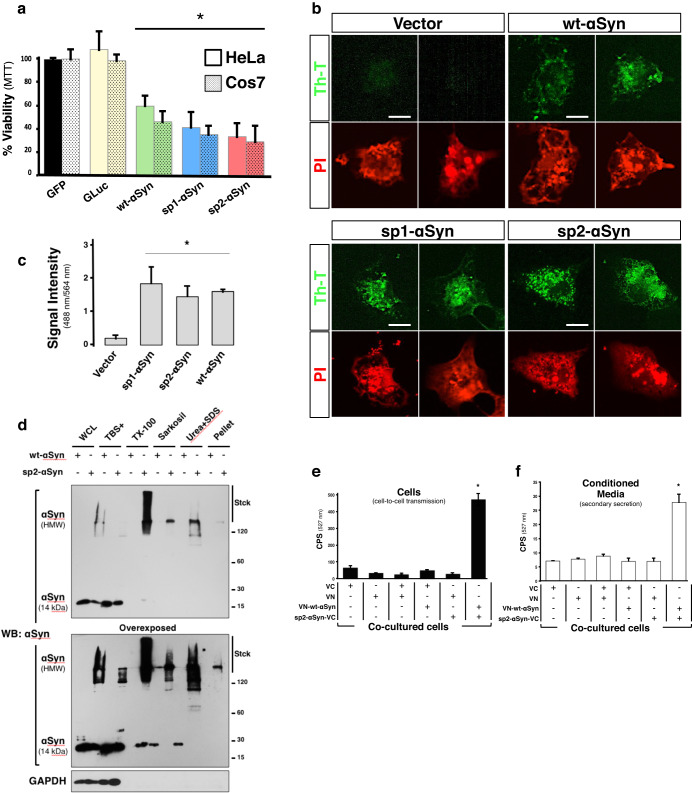
sp2-αSyn is toxic, amyloidogenic and cell-to-cell transmitted. **a** Determination of cell viability of HeLa and Cos7 cells expressing GFP, the luciferase of *Gaussia princeps* (GLuc), wt-, sp1- or sp2-αSyn by the MTT assay. Data is shown as the media ± SD. *p < 0.05 compared to GFP-expressing cells (one-way ANOVA followed by the post hoc Dunnett’s test, *n* = 5). **b** Representative confocal microscopy images of Cos7 cells expressing wt-, sp1- or sp2-αSyn and stained with the amyloid-specific dye thioflavin-T (Th-T). Cells transfected with an empty vector (Vector) were used as a negative control. Scale bar: 10 μm. **c** Quantification of Fig. 2b. *p < 0.05 (one-way ANOVA followed by the post hoc Tukey’s test, *n* = 5). **d** WCL of Cos7 cells expressing wt- or sp2-αSyn were subjected to sequential extraction by detergents and subsequently analyzed by WB. **e**, **f** HeLa cell clones expressing either VN or VC (N- and C- terminus half of the Venus fluorescent protein, respectively), or the fusion proteins VN-wt-αSyn or sp2-αSyn-VC were co-cultured as indicated. 48 h later the cells were harvested and fluorescence was quantified in living cells (**e**) or in the conditioned media to asses secondary secretion (**f**). Data is shown as the media ± SD. CPS counts per second. *p < 0.005 (one-way ANOVA followed by the post hoc Tukey’s test, *n* = 5).

We next studied cytotoxicity, and transiently expressed wt-αSyn, sp1-αSyn, or sp2-αSyn in Cos7 and HeLa cells. We found a significant reduction of cell viability in both cell types upon expression of either wt-αSyn, sp1-αSyn, or sp2-αSyn 48 h post-transfection (Fig. 2a). On the contrary, no effect on cell survival was observed in cells expressing eGFP or the protein that naturally contain sp2, the luciferase of *Gaussia princeps* (GLuc), indicating that the observed toxicity was attributable to αSyn and not to the signal peptides or to increased protein expression or secretion. We next studied whether overall secretion is impaired in sp1-αSyn and sp2-αSyn expressing cells, and carried out experiments where the secreted reporter enzyme *Gaussia princeps* luciferase was co-expressed in mammalian cells together with sp1- and sp2-αSyn. Because *Gaussia princeps* luciferase is secreted to the media, the luciferase activity in the CM positively correlates with the levels of the secreted enzyme. As shown in Supplementary Fig. 2d, no differences in *Gaussia princeps* secretion were observed in cells expressing sp1- or sp2-αSyn. The luciferase activity was similar to cells expressing wt-αSyn as well as a control-secreted protein called sp2-VC. The data thus indicates that secretion is not substantially impaired when sp1- or sp2-αSyn are expressed in the cells.

We then asked whether the secreted αSyn could form higher-order structures such as amyloid oligomers, fibrils, and aggregates, as suggested by the experiments shown in Fig. 1b. We found that cells expressing the secreted variants as well as the wild-type protein were stained positively with thioflavin-T, a dye that positively stains amyloids (Fig. 2b, c).

Because the sp2 signal peptide was efficiently cleaved off from the fusion protein, while sp1- was not (Fig. 1d), only sp2-αSyn was used in subsequent experiments. The cells expressing sp2-αSyn were immunolabeled with antibodies characteristic of Lewy bodies in PD such as anti-αSyn phosphorylated at serine 129, anti-P62, and anti-ubiquitin (Supplementary Fig. 2e). Phosphorylation at serine 129 was confirmed by Western blot, which showed that αSyn phosphorylation is increased in sp2-αSyn-expressing cells (Supplementary Fig. 2f). It also showed a delayed mobility in SDS gels for modified αSyn that, together with ubiquitination and aggregation, explains the HMW bands immunoreactive for αSyn in sp2-αSyn-expressing cells. In this sense, we confirmed the formation of higher-order structures by biochemical analyses on wt- and sp2-αSyn, which revealed that the HMW species of αSyn formed by sp2-αSyn in mammalian cells are greatly insoluble, as they were extracted with detergents only partially (Fig. 2d). Although wt-αSyn could also form insoluble HMW species, which were extracted with mild treatments, the data indicated that the aggregates formed by sp2-αSyn are more stable and abundant. Altogether the data indicated that sp2-αSyn is phosphorylated, ubiquitinated, and forming protein aggregates inside cells. Important for the interpretation of the data is that cells transfected with plasmids encoding wt- or sp2-αSyn are kept for 48 h in culture in order to obtain high levels of the ectopically expressed protein. During this period of time, two non-mutually exclusive processes take place; αSyn release and uptake, and αSyn aggregation can occur in any of these processes.

Next, we investigated whether the released αSyn could be cell-to-cell transmitted, and used a bimolecular fluorescence complementation (BiFC) approach based on the homotypic physical interaction between αSyn monomers to form multimers, oligomers, and higher-order aggregates (Supplementary Fig. 2g). The BiFC approach allows monitoring not only αSyn transcellular spreading but also intermolecular seeding (templated aggregation). We expressed in mammalian Cos7 cells the N- or C-terminal halves of the Venus fluorescent protein (VN and VC, respectively), and the fusion proteins VN-wt-αSyn (VN fused to the N-terminal end of wt-αSyn) and sp2-αSyn-VC (VC fused to the C-terminal end of sp2-αSyn). Cells expressing each individual protein for 48 h were cultured alone or co-cultured, and fluorescence was quantified in live cells 24 h later. The high fluorescence signal was obtained when cells expressing sp2-αSyn-VC were co-cultured with those expressing VN-αSyn (Fig. 2e), indicating that the released αSyn is cell-to-cell transmitted and able to physically interact with VN-wt-αSyn in neighbor cells. No fluorescence was detected in any other experimental condition assayed, indicating that reconstitution of the full-length Venus protein resulted from the specific physical interaction between wt-αSyn and sp2-αSyn. This was confirmed by confocal microscopy that showed strong co-localization of αSyn and Venus in VN-wt-αSyn acceptor cells that had been co-cultured with cells expressing sp2-αSyn-VC (Supplementary Fig. 2h, i). Quantification of fluorescent cells (counts) by confocal microscopy revealed that very few but still detectable fluorescent cells can be obtained by expressing αSyn fused to any of the GFP fragments (VC or VN). It also showed that wt-αSyn is cell-to-cell transmitted (Supplementary Fig. 2h). Excluding the possibility of sp2-αSyn-VC transmission by direct cell-to-cell contact, conditioned media containing this protein was also able to imprint a reconstituted Venus protein on the respective acceptor cells (Supplementary Fig. 2j). Due to natural secretion, CM containing the wild type protein was also able to reconstitute Venus in sp2-αSyn-VC acceptor cells.

We then investigated the ability of the formed sp2-αSyn-VC/VN-wt-αSyn species to be released by secondary secretion^41^ and determined fluorescence in freshly collected CM of the co-cultures (see the “Methods” section). As before, fluorescence was observed only in CM of co-cultured sp2-αSyn-VC and VN-wt-αSyn-expressing cells (Fig. 2f). Supporting the αSyn homotypical interaction, WB analyses indicated that these fusion proteins form stable HMW species (Supplementary Fig. 2k), although they were different than the species formed by sp2-αSyn probably due to a slower aggregation by the VC fragment in sp2-αSyn-VC. From these analyses, we concluded that a removable functional short signal peptide induced a robust secretion of αSyn, which is found as toxic amyloids and stable HMW species that are cell-to-cell transmitted and seeding compatible. Thus, sp2-αSyn fulfills the major requirements for a prion-like protein.

### Development of a novel mouse model of PD with enhanced αSyn secretion

The distinctive behavior of sp2-αSyn prompted us to develop a novel mouse model of Parkinson’s disease based on enhanced αSyn secretion and cell-to-cell transmission. The model was established by expressing sp2-αSyn in midbrain neurons of wild-type non-transgenic C57BL/6J mice. Ectopic expression of sp2-αSyn in the mouse brain was carried out by a single administration of an adeno-associated viral vector (AAV) encoding this protein. For comparison and in order to assign the brain responses triggered specifically by the secreted variant, wt-αSyn was expressed similarly. Moreover, an empty AAV or an AAV encoding EGFP was used as a control for viral transduction and ectopic protein expression, respectively.

**Fig. 3:**
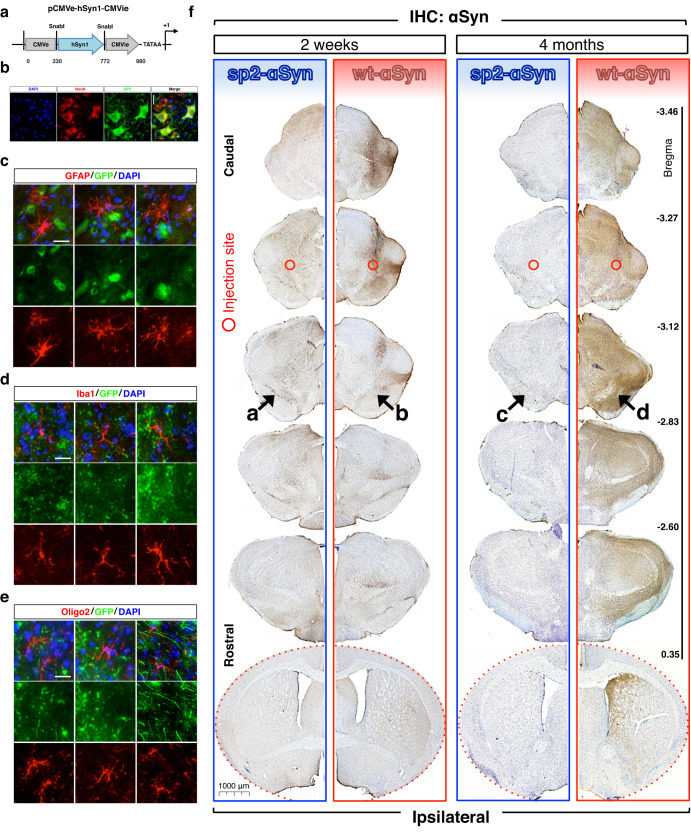
Neuronal expression of secreted αSyn in the mouse brain. **a** Schematic representation of pCMVie-hSyn-CMVe, the neuron-specific promoter used for targeted expression of the aSyn variants in wild-type mice. Target site (present in CMV but destroyed in the final construct) of the restriction enzyme SnabI used to generate the hybrid promoter is shown. **b**–**d** Representative immunofluorescence images of brain sections from wild-type mice transduced with an AAV9 carrying the eGFP gene under the control of the pCMVie-hSyn-CMVe promoter. A remarkable co-localization of eGFP with NeuN-positive neurons (**b**) together with the absence of fluorescence in all three types of glial cells assayed (**c**, and **d**) confirms the specificity of this promoter toward neurons. While neurons were immunolabeled with anti-NeuN antibodies (in red) (**b**), Astrocytes, microglia and oligodendrocytes were stained with anti-GFAP (**c**), anti-Iba1 (**d**) and anti-Oligo2 (**e**) antibodies (all in red), respectively (**b**). Scale bars: 50 μm. **f** Representative immunohistochemistry (IHC) images of consecutive coronal sections of forebrains of wild-type non-transgenic mice transduced with sp2-αSyn (*n* = 8, blue) and wt-αSyn (*n* = 8, red) AAVs for 2 weeks and 4 months. Approximate rostro-caudal coordinates taking the bregma as reference are indicated on the right. IHC was carried out with the anti-αSyn antibody clone LB509 which recognizes humans but not murine αSyn. Only hemispheres where the viral vector was injected (ipsilateral) are shown. Injection site is indicated with open red circles. Arrows, substantia nigra. Scale bar: 1000 μm.

Targeted expression of sp2-αSyn in the mouse brain was driven by a novel promoter called pCMVie-hSyn-CMVe (Fig. 3a), that when used in combination with AAV serotype 9 (AAV9), induced a robust and cell type-specific expression of the transgenes in neurons, as shown by co-localization analyses using the neuronal marker NeuN and an eGFP reporter gene under the control of pCMVie-hSyn-CMVe (Fig. 3b). Confirming that ectopic expression is restricted to neurons, co-localization studies using the GFAP, Iba1, and oligo2 glia-specific markers revealed that the transgene (eGFP in this case) is not expressed in astrocytes, microglia or oligodendrocytes (Fig. 3c–e). Expression of sp2-αSyn was achieved by a single unilateral stereotaxic injection of the AAV9 in the deep mesencephalic nucleus (DMN), a brain structure with afferent projections to the ipsilateral motor and somatosensory cortex, the superior colliculus and substantia nigra. In order to evaluate the effect of sp2-αSyn over time, animals were sacrificed 2 weeks or 4 months post-AAV9 injection (w.p.i. and m.p.i, respectively). We first carried out immunohistochemistry (IHC) analyses of consecutive coronal brain sections (bregma −3.46 to +0.35) using an anti-αSyn antibody that recognizes human αSyn but not the αSyn of the host (mouse) (clone LB509^42^). Using this antibody, only the ectopically expressed wt- and sp2-αSyn can be observed in the IHC. These experiments revealed that at 2 w.p.i. a substantial accumulation of αSyn was observed in the ipsilateral hemisphere of animals expressing either wt- or sp2-αSyn (Fig. 3f). The extent and distribution of the αSyn immunoreactivity was, however, markedly different in wt- and sp2-αSyn-expressing animals. In brains expressing sp2-αSyn for 2 weeks, αSyn staining was abundant near the injection site at the DMN and also in the substantia nigra (SN) and adjacent areas. Very low levels of immunoreactivity for αSyn were found in the striatum and cortex, while it was absent in the hippocampus of these animals (Fig. 3f and Supplementary Fig. 3a). A much more robust αSyn immunoreactivity was observed in brains expressing wt-αSyn at 2 w.p.i. At this time point, the αSyn-specific staining was massively found near the injection site at the DMN and other areas of the midbrain such as SN, ventral tegmental area (VTA), superior colliculus, and certain areas of the thalamus such as the ventral posterolateral thalamic nucleus (Fig. 3f).

With the exception of the substantia nigra and adjacent areas such as the VTA, in sp2-αSyn-expressing mice, the overall extent and topographical distribution of the staining decreased over time. Compared to animals observed at 2 w.p.i., a lower αSyn signal was observed in most brain structures of animals expressing sp2-αSyn for 4 months (Fig. 3f). Strikingly, and despite the substantial reduction of human αSyn levels in extranigral structures, animals observed at 4 m.p.i. displayed similar staining of αSyn in the SN and VTA, compared to animals observed at 2 w.p.i. In contrast to sp2-αSyn, IHC analyses on mice expressing wt-αSyn for 4 months revealed that the number and topographical distribution of the αSyn immunoreactivity dramatically increased over time. Indeed, at 4 m.p.i. ectopic αSyn was massively found not only in the midbrain but also in the striatum, hippocampus, and cortex as well as in the contralateral hemispheres (Fig. 3f and Supplementary Fig. 3a).

The control groups corresponding to animals injected with an empty AAV9 or with an AAV9 carrying the eGFP gene showed no αSyn immunoreactivity at any time assayed (Supplementary Fig. 3b). Likewise, no αSyn staining was observed in the brain of animals with robust αSyn immunoreactivity when the primary antibody was omitted in the IHC, confirming the specificity of the staining (Supplementary Fig. 3c).

One of the main advantages of AAV9 is that it cannot self-propagate within the brain unless a second “helper” virus is added to the system. Therefore, the expression of the transgene is limited only to the transduced cells. To rule out the possibility of a retrograde/anterograde transport of the AAV9 infective particle used to express sp2-αSyn in the mouse brain, we analyzed the substantia nigra of mice injected with an AAV9 carrying the eGFP gene. The data indicates that anterograde/retrograde transport is not occurring in these mice, as no fluorescence was detected in the SN of these mice (Supplementary Fig. 3d). This is also supported by the fact that the pathology elicited by wt-αSyn shows a completely different distribution compared to sp2-αSyn. Thus, the data overall indicated that while wt-αSyn displayed a widespread distribution that increased over time, sp2-αSyn was found only in selected neuroanatomical areas of the brain, which at later time points are only the SN and in minor extents the VTA. The brain distribution of sp2-αSyn accumulation is not due to anterograde transport of the viral vector used for transduction.

### Secreted αSyn triggers a robust LB-like pathology restricted to the substantia nigra

**Fig. 4:**
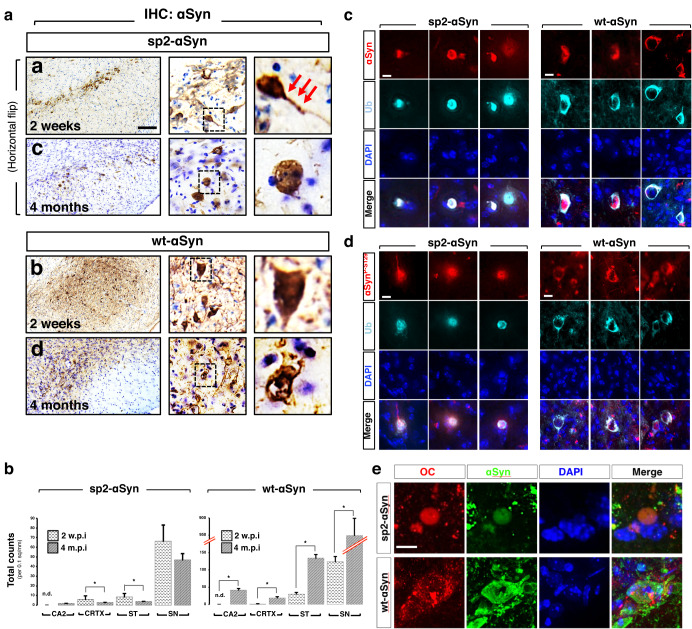
Lewy body-like pathology elicited by secreted αSyn. **a** Higher-power-magnified IHC images of the substantia nigra of wild-type mice transduced with AAV9 encoding sp2-αSyn or wt-αSyn for 2 weeks and 4 months. Horizontal flip of images from sp2-αSyn (panels **a** and **c**) are shown. IHC was carried out with the anti-αSyn antibody LB509 which only recognizes human αSyn. Arrows indicate inclusions present in neuronal projections. Scale bar: 10 μm. **b** Quantification of the αSyn-positive inclusions in the CA2 hippocampus (CA2), cortex (CRTX), striatum (ST), and substantia nigra (SN) of mice transduced with AAV-sp2-αSyn (right) or AAV-wt-αSyn (left) for 2 weeks (2 w.p.i.) or 4 months (4 m.p.i.). * *p* < 0.05 (unpaired, two-tailed distribution Student’s *t*-test, *n* = 5). n.d. non-detected. Data is shown as the media ± SD. **c**–**e** Representative double immunofluorescence images of mice expressing sp2-αSyn or wt-αSyn for 4 months. Immunostaining was carried out with antibodies specific for ubiquitin (Ub) (**c**), αSyn phosphorylated at serine 129 (**d**), or the OC antibody that recognizes amyloid oligomers and fibrils (**e**). DAPI was used for nuclear staining. Scale bar, 5 μm (**c**, **d**), and 10 μm (**e**).

A closer examination of the αSyn immunoreactivity revealed that in either wt- and sp2-αSyn-expressing animals αSyn mostly accumulated in well-defined structures present within cell bodies and neuronal projections (Fig. 4a). Some of these structures are round-shaped and perinuclear. The subcellular localization as well as the morphology of these structures suggested the presence of Lewy body-like inclusions, macroscopic structures that characterize several animal models of Parkinson’s disease^20^. Supporting this idea, these inclusions were positive for ubiquitin and phosphorylated αSyn at serine 129, two histological hallmarks of Lewy bodies (Fig. 4c, d and Supplementary Fig. 4a, b). Moreover, inclusions formed by either wt-αSyn or sp2-αSyn were both immunolabeled with an antibody that recognizes amyloid fibrils (Fig. 4e). The remarkable differences in the extent, distribution and temporal progression of the pathology elicited by sp2-αSyn and wt-αSyn (Fig. 3) was accompanied by a distinctive morphology of the inclusions; inclusions formed by the secreted αSyn variant were mainly perinuclear round-shaped structures while the ones formed by wild-type αSyn were diffuse and distributed throughout the cell body (Fig. 4a, c–e). When expressed in mouse brains, the secreted αSyn variant formed much more abundant HMW species that resisted denaturation by high temperatures, detergents, and denaturing agents such as urea, as indicated by WB (Supplementary Fig. 4c, d). Thus, the data indicated that neuron-derived extracellular αSyn suffices LB-like pathogenesis in wild-type non-transgenic mice. Moreover, it showed that the pathology elicited by sp2- and wt-αSyn is markedly different in terms of inclusion morphology and brain distribution.

### The substantia nigra is particularly susceptible to secreted αSyn

**Fig. 5:**
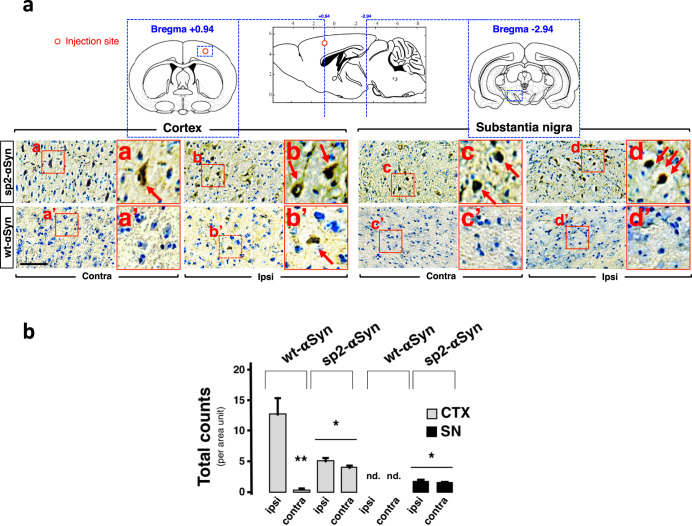
αSyn spreading in the mammalian brain. **a** Representative IHC images of coronal brain sections of cortex and substantia nigra of wild-type mice that received a single injection of an AAV9 encoding sp2- or wt-αSyn in the cortex. Animals were sacrificed at 4 months post-surgery. In the upper part a schematic representation of the anatomical structures analyzed that includes approximate rostro-caudal coordinates with the bregma as reference. Quantification is shown in (**b**). Data is shown as total counts per area unit ± SD. Contralateral (Contra) and ipsilateral (Ipsi) hemispheres are shown. Scale bar: 200 μm. *p < 0.05 compared to wt-αSyn. **p < 0.05 compared to ipsi (one-way ANOVA followed by the post hoc Tukey’s test).

The fact that in sp2-αSyn-expressing mice the pathology observed at 4 m.p.i. was restricted to the SN suggested that nigral neurons are highly susceptible to the αSyn that is released by the neurons of the host. We next tested this interesting hypothesis by expressing sp2-αSyn in a brain structure distant to the SN such as the motor cortex M1. Ectopic expression was carried out in wild-type non-transgenic mice by a single unilateral stereotaxic injection of the AAV9 vectors, and as before, animals expressing wt-αSyn in the same cortical structures were included for comparative purposes. We readily observed LB-like inclusions within the SN of animals expressing sp2-αSyn in the motor cortex for 4 months (Fig. 5a, b). As expected for a secreted αSyn variant reaching midbrain structures via the extracellular milieu, inclusions were found in both the ipsilateral and the contralateral SN, as well as in other neuroanatomical areas such as the striatum and hippocampus (Supplementary Fig. 5a). αSyn pathology in the SN was much less pronounced compared to mice that had received the AAV9s directly in the DMN of the midbrain (Figs. 3 and 4). Importantly, αSyn pathology was absent in the SN of animals that expressed wt-αSyn in the cortex at this time point. As expected, inclusions were observed near the injection site in cortical structures of animals expressing either sp2-αSyn or wt-αSyn. Thus, we confirmed that nigral neurons are particularly susceptible to neuronal extracellular αSyn.

### Secreted αSyn triggers neuronal death and neuroimmflamation

**Fig. 6:**
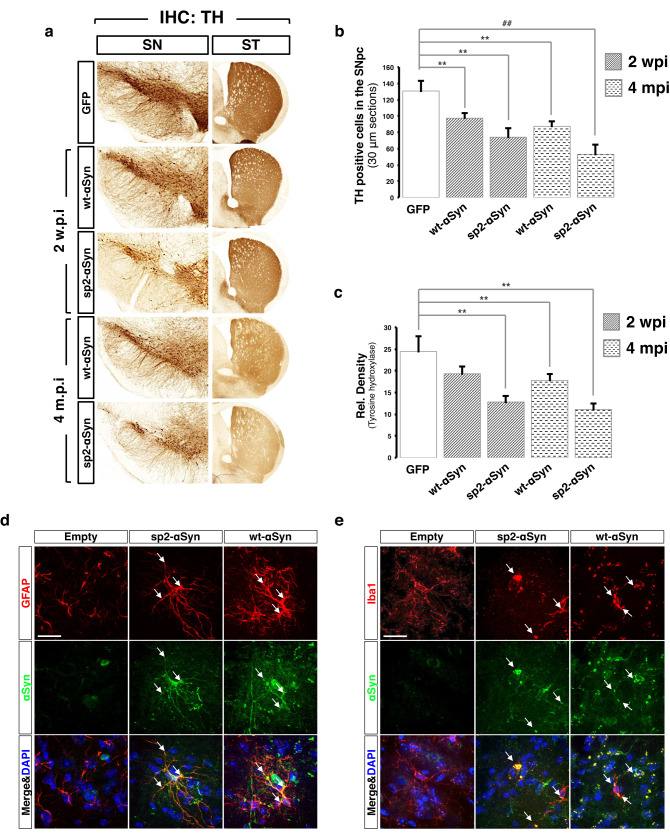
Secreted αSyn triggers neuronal death and neuroimmflamation. **a** Representative IHC images of coronal sections of the substantia nigra (SN) and striatum (ST) of mice injected wt- (*n* = 5) or sp2-αSyn (*n* = 6) AAVs for 2 weeks and 4 months. Animals transduced with AAV-GFP for 4 months (*n* = 5) were used as controls (upper part). IHC was carried out with an anti-tyrosine hydroxylase (TH) antibody. **b** and **c** Quantification of the number of TH-positive cells (**b**) or TH signal density (**c**) from the substantia nigra of mice transduced with AAV-sp2- (*n* = 6) or AAV-wt-αSyn (*n* = 5) for 2 weeks or 4 months. Animals expressing GFP (*n* = 5) for 4 months were used as controls. Data is shown as the media ± SD. **p < 0.05 (one-way ANOVA followed by the post hoc Tukey’s test). **d**, **e** Double immunofluorescence of the substantia nigra of mice transduced with AAV-GFP, AAV-sp2-αSyn, or AAV-wt-αSyn for 4 months. Human αSyn (green) was immunostained together with the marker for astrocytes GFAP (**d**) or microglia Iba1 (**e**) (both in red). Arrows indicate co-localization between the glial marker and αSyn in astrocytes or microglia. DAPI was used for nuclear staining. Scale bar: 50 μm.

We then asked whether the secreted αSyn could lead to neuronal loss and neuroimmflamation. To this end brain slices of mice transduced with the eGFP, sp2-αSyn, and wt-αSyn AAVs for 2 weeks and 4 months were subjected to IHC analyses, that revealed that both αSyn variants elicited a similar and significant (*p* < 0.05) loss of tyrosine hydroxylase (TH)-positive neurons with the concomitant appearance of dystrophic neurites in the SN (Fig. 6a). This was accompanied by a comparable decrease of TH-positive nerve terminals in the striatum. Cell loss was progressive; at 2 w.p.i. and 4 m.p.i. the decrease in cell number was around 20% and 40%, respectively (Fig. 6b, c). In order to evaluate neuroinflammation in response to the different αSyn variants, we analyzed the profiles of astroglial and microglial activation in animals expressing sp2-αSyn and wt-αSyn for 4 months. Brain slices subjected to double immunofluorescence analyses with antibodies against human αSyn and the astrocyte-specific protein glial fibrillary acidic protein (GFAP) revealed that astrocytes of sp2-αSyn and wt-αSyn expressing animals displayed enlarged bodies and altered ramifications, compared to mice expressing eGFP (Fig. 6d and Supplementary Fig. 6a). The morphological changes induced by both αSyn variants corresponded to type B and are indicative of astrocyte activation^43^. Activated astrocytes were enriched in areas of high αSyn expression in both groups of animals (Supplementary Fig. 6a). Moreover, αSyn and GFAP co-localized in well-defined intracytoplasmic foci in these mice, although co-localization was higher in sp2-αSyn expressing brains (Fig. 6d and Supplementary Fig. 6a). Likewise, double immunofluorescence for αSyn and the microglia-specific protein ionized calcium-binding adapter molecule 1 (Iba1) indicated marked differences in the morphology of microglia of mice expressing eGFP and the two αSyn variants (Fig. 6e and Supplementary Fig. 6b). Compared to controls, microglia of either sp2-αSyn or wt-αSyn expressing mice displayed enlarged cell bodies with pseudo-ameboid shape and contracted ramifications. These morphological changes were mainly observed in areas of high αSyn expression (Fig. 6e and Supplementary Fig. 6b). Two stages of microglia activation were quantified according to previous works^43^; while mice expressing eGFP contained type-A resting microglia, mice expressing αSyn were enriched in B and C types corresponding to exacerbated microglial activation. Like GFAP, αSyn co-localized with Iba1 in cell bodies and ramifications of microglia of αSyn expressing mice. Noteworthy, as AVV9-mediated expression of αSyn was targeted to neurons by the neuronal pCMVie-hSyn-CMVe promoter, and glia express very low levels of αSyn, the presence of high levels of αSyn in activated glia accounts for the neuron-to-glia transmission of this protein in these mice. Confirming neuroinflammation, mice expressing the secreted αSyn variant displayed increased levels of GFAP and Iba1 (Supplementary Fig. 6c) as shown for activated glia in animal models of neurodegeneration.

### Secreted αSyn triggers a Parkinson’s disease-like pathology in wild-type non-transgenic mice

**Fig. 7:**
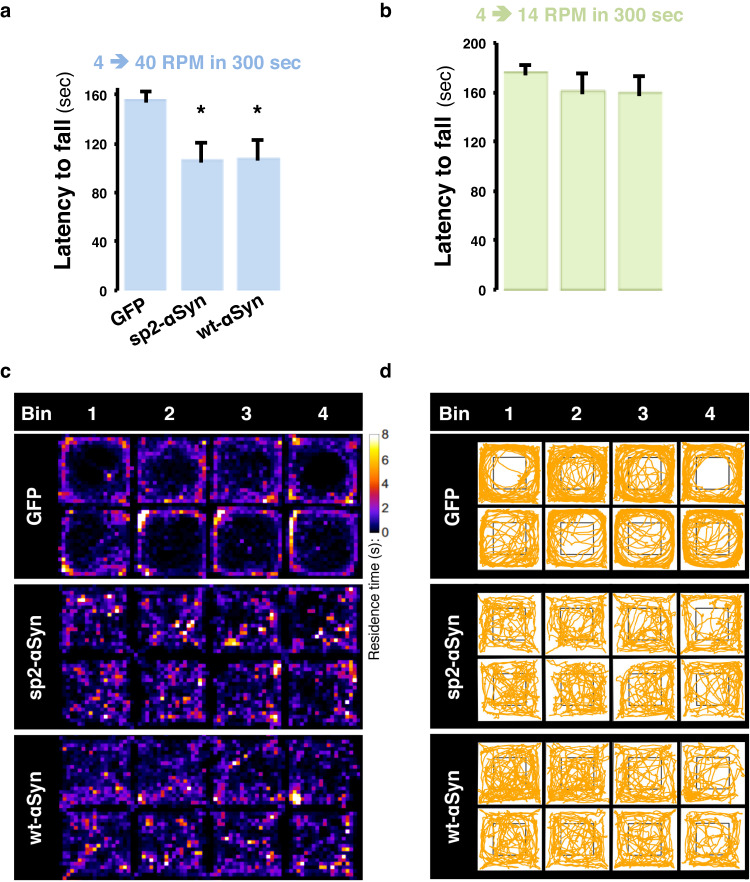
Locomotor deficits elicited by secreted αSyn. **a** and **b** Mice (*n* = 6) of each group were assayed by an accelerating rod that started at 4 RPM and reached 14 RPM (**a**) or 45 RPM (**b**) in 300 s. Data is shown as the latency to fall (calculated as the averaged time in seconds to fall)±SD. *p < 0.05 (one-way ANOVA followed by the post hoc Dunnett’s test). **c**, **d** Representative heat maps of residence time (**c**) and traje**c**tory plots (**d**) derived from the open field test within a 20 min session divided into four consecutive sessions (bins) of 5 min each. Two representative maps from mice transduced with eGFP (*n* = 6), sp2-αSyn (*n* = 6), or wt-αSyn (*n* = 5) AAVs for 4 months are shown.

We then assessed locomotor activity, motor performance, and anxiety-like phenotypes using the rotarod and open field tests in animals expressing the αSyn variants for 4 months. Animals expressing either sp2-αSyn or wt-αSyn failed the rotarod test when a mild acceleration (4–40 RPM in 300 s) was applied to the rod, as shown by a significantly (*p* < 0.05) reduced latency to fall (Fig. 7a). That animals did not display abnormalities in a slow accelerating (4–14 RMP in 300 s) rod indicated absence of major motor defects (Fig. 7b), which is expected for a Parkinson’s disease-like phenotype in rodents. In agreement with impaired locomotor activity, quantification of the trajectories of the open field test revealed that the distance traveled by animals expressing both αSyn variants was similar, but significantly (*p* < 0.05) reduced by ~50% when compared to eGFP-expressing mice (Fig. 7c, d and Supplementary Fig. 7a). Moreover, in control mice the occupancy of the center zone was <10% of the total time registered, whereas animals expressing either sp2-αSyn or wt-αSyn stayed within this area around 30% of the time (Fig. 7c, d and Supplementary Fig. 7b). Likewise, both groups of animals showed significant (*p* < 0.05) differences in the distance traveled within the center zone as the αSyn mice traveled twice than control animals (Supplementary Fig. 7c). Thus, by displaying a nigral αSyn pathology, neuronal loss, neuroinflammation, and motor deficits, we conclude that sp2-αSyn induced a Parkinson’s disease-like pathology in non-transgenic wild type mice.

## Discussion

The cell-to-cell transmission of αSyn via cycles of secretion and uptake is believed to be a crucial process in the pathogenesis and spreading of Lewy bodies and alike^44,45^. It underlies the progression of a selective spectrum of human neurodegenerative diseases known as synucleinopathies. The transcellular spreading of αSyn can be recapitulated in vivo by administration of exogenous material such as brain homogenates or αSyn fibrils produced with recombinant proteins expressed in heterologous systems^20,46–53^. Depending on the composition of the injected material, the site of administration, and the genetic background of the recipient, exogenous αSyn can be internalized by brain cells triggering an LB-like pathology and eventually a Parkinson’s disease-like phenotype^20,54^. Despite models based on the administration of exogenous material are very useful to elucidate key aspects of αSyn transmission, they preclude investigations on the extracellular αSyn released by the neurons of the host and how it affects the mammalian brain. In order to overcome this limitation, here we developed the first animal model with increased cell-to-cell αSyn transmission based on enhanced secretion of neuronal αSyn. The model recapitulates the cell-to-cell transmission of αSyn in the mammalian brain where pathological species of αSyn are released by the afflicted neurons that harbor LB/LNs. Experiments with cultured cells suggested that, in these mice, neuron-derived extracellular αSyn is internalized by healthy neighbor cells to recruit the soluble counterpart of recipient cells promoting the formation of Lewy bodies. The model is supported by previous works showing that neuronal αSyn is released via classical secretion^55^, non-conventional secretion^35,56^, or exosomes^21^. αSyn release via the secretory pathway is further supported by the interaction of αSyn with proteins of the ER and Golgi apparatus in PD and related pathologies^57–60^. HMW species of αSyn are found along the secretory pathway in PD and animal models of synucleinopaties^61–64^. Moreover, like sp2-αSyn, the αSyn contained in secretory vesicles is more neurotoxic and prone to aggregation than the cytoplasmic counterpart^37,56^. Of note, it has been shown that overexpression of certain ER and Golgi proteins suppresses αSyn cytotoxicity^57,65^. This, together with the fact that αSyn is targeted to the secretory pathway as part of its normal life cycle in yeast, where this pathway is essential for αSyn cytotoxicity^66^, again hinting at an important role of secretion in αSyn pathophysiology.

Reminiscent of the αSyn contained in LBs and LNs, the secreted αSyn variant described in this work accumulated as stable HMW species that triggered the major phenotypic changes that define Parkinson’s disease. It included an αSyn pathology mainly restricted to the substantia nigra, raising the fundamental question of why nigral neurons are particularly susceptible to deposition of the cell-to-cell transmitted αSyn, while other areas of the brain are refractory to it. This remarkable susceptibility also calls for the simplistic idea of a non-cell autonomous mechanism as responsible for nigral degeneration in Parkinson’s disease to be revisited^67,68^. Accumulation of LB-like inclusions mainly in the substantia nigra is rarely observed in non-transgenic mice injected with synthetic pre-formed αSyn fibrils^20,47,69,70^. Likewise, ectopic expression of wt-αSyn in the brain of non-transgenic mice triggers a robust and widespread pathology that largely exceeds nigral structures (this work and refs. ^71,72^). Although the reasons for the observed differences between wt- and sp2-αSyn are still enigmatic, the data suggested that a biochemical imprinting is acquired by αSyn along the secretory pathway. In this sense, the specific environments of the different secretory compartments might affect αSyn in several ways. Protein aggregation along the secretory pathway is a common process that regulates the physiology and pathology of many functional and pathological aggregation-prone proteins, as exemplified by the big family of peptide hormones^73^. Protein aggregation is massively induced in early secretory compartments by increased expression levels^74^, a phenomenon of clinical relevance in familial Parkinson’s disease with increased αSyn levels due to the multiplication of *SCNA*, the gene encoding αSyn in humans^7,75^. High calcium concentration^76,77^ and low pH^78^, all of them found along the secretory pathway, are known not only to promote αSyn aggregation but also its fragmentation^79^ and the formation of structurally different αSyn aggregates or strains with increased seeding and cytotoxic properties for cells and animals. Post-translational modifications that take place exclusively in the secretory pathway such as O-glycosylation have been reported for αSyn. Indeed, αSyn has been shown to be *O*-glycosylated in autosomal-recessive Parkinson’s disease patients^80^. Moreover, the ER acetylation machinery specifically counteracts toxic αSyn aggregates that form within the secretory compartments but not those that form in the cytosol^81^. Finally, the different protein repertoires of the cytosol and secretory compartments would result in different αSyn molecular interactions that might promote cytotoxicity.

Regardless of the biochemical imprinting acquired along the secretory pathway, that the secreted αSyn accumulates in the substantia nigra is of critical clinical importance as it phenocopies PD and therefore could give crucial hints on disease pathogenesis, progression, and therapeutic intervention. The extent of the stereotypical brain expression of the control gene eGFP (Supplemental Fig. 4d), as well as wt-αSyn, indicates that sp2-αSyn is expressed and released by cells of several neuroanatomical structures. Although absolute quantification of the levels of the ectopic expression of sp2- and wt-αSyn was not included in this study, the data indicate that sp2-αSyn preferentially accumulates in nigral structures and this is enhanced at 4 m.p.i. At least two mechanisms could explain these findings: (i) while nigral neurons are capable of internalizing extracellular αSyn, other cell types are not; and (ii) all cells are capable of internalizing and degrading αSyn except nigral neurons, which internalize αSyn but are less efficient at degrading it. Ongoing experiments aimed at elucidating why nigral neurons are particularly susceptible to the accumulation of sp2-αSyn will shed light on the mechanisms involved in the uptake and the clearance of neuronal extracellular αSyn.

The novel mouse model presented here reinforces the idea that αSyn cell-to-cell propagation plays a critical role in synucleinopathies. The model is therefore useful for elucidating the mechanism of disease progression and αSyn cell-to-cell transmission in vivo, and we anticipate that it will guide the discovery of novel therapeutics for Parkinson’s disease and related synucleinopathies.

## Methods

### Cell culture

Cos7, SH-SY5Y, HeLa, and Hek-293 cells were purchased from ATCC and cultured in Dulbecco’s modified Eagle medium (DMEM, pH 7.3) supplemented with 10% fetal calf serum, 4 mM l-glutamine, 100 U/ml penicillin and 100 mg/ml streptomycin. Only undifferentiated SH-SY5Y cells were used in this study. Treatment with the different inhibitors was carried out in 2% fetal calf serum. Monensin was used at a final concentration of 2 µM for 4 h. The MTT assay (CellTiter 96 Non-Radioactive Cell Proliferation Assay, Promega) was performed according to the manufacturer’s instructions.

### Plasmids and cloning

Plasmid construction and DNA manipulations were performed following standard protocols. The DNA *sequences* of *all constructs* were verified by *sequencing prior to use*. The pCMVie-hSyn-pCMVe promoter was obtained by amplifying by PCR a 419 bp fragment of the human Synapsin-1 (hSyn) promoter contained in the pLV-Syn-pri132 vector using the primers: forward, TA**GATATC**CTGCAGAGGGCCCTGCGTAT, reverse TA**GATATC**TCTCGACTGCGCTCTCAGG (EcoRV restriction sites are indicated bold), and inserting it into the human cytomegalovirus (CMV) promoter of the plasmid pcDNA3.1+ linearized with SnaBI. The resulting plasmid, called pcDNA3-CMV-hSyn-CMV was digested with NheI and HindIII and used to generate expression vectors by insertion of PCR fragments containing the coding regions of eGFP, wt-αSyn, sp1-αSyn, and sp2-αSyn. The primers used for such PCRs were: eGFP forward, TTA**GCTAGC**ATGGTGAGCAAGG, reverse, TCC**AAGCTT**TCATTACTTGTACAGCTCG wt-αSyn forward, TTA**GCTAGC**ATGGATGTATTC ATG AAAGGACT, reverse, TCC**AAGCTT**TCATTAGGCTTCAGG; Sp1-αSyn forward, TTA**GCTAGC**ATGGAGTTTGGGCTGAGCTGGGTTTTCCTCGTTGCTCTTTTTAGAGGTGTCCAGTGTGATGTATTCATGAAAGGACT, reverse, TCC**AAGCTT**TCATTAGGCTTCAGG; sp2-αSyn forward, TTA**GCTAGC**ATGGGAGTCAAAGTTCTGTTTGCCCTGATCTGCATCGCTGTGGCCGAGGCCGATGTATTCATGAAAGGACT, reverse, TCC**AAGCTT**TCATTAGGCTTCAGG, (NheI and HindIII restriction sites are indicated bold).

To generate the vectors for AAV9 production, AvrII and SnaBI restriction sites were inserted into the 5’ end of the promoter of the pcDNA3-CMV-hSyn-CMV-eGFP vector. This was carried out by digestion with MluI and BglII (located at 5’ of CMVie-hSyn-CMVe) and insertion of a dsDNA oligonucleotide generated with the annealed oligos: GATCTTACGTACCTAGGA and CGCGTCCTAGGTACGTAA. Next, a PCR product containing the Woodchuck Hepatitis Virus (WHP) Posttranscriptional Regulatory Element (WPRE) generated with the primers: forward, ATA**GCGGCCGC**TCCGAATCAACC, reverse, ATA**GCGGCCGC**GGGAGGCGGCCCAAAGGG; (NotI restriction sites are indicated bold), was digested and inserted into the NotI sites of this vector. Finally, the cassette containing CMVie-hSyn-CMVe, eGFP, WPRE, and bGH polyA was excised by digestion with AvrII and PvuII (the latter located at 3’of bGH polyadenylation site), blunted with DNA polymerase I large (Klenow) fragment and ligated into the pAAV-MCS vector digested with NotI and blunted with Klenow. The resulting vector, called pAAV-CMV-hSyn-CMV-eGFP-WPRE (subsequently used to produce the AAV9-eGFP viral particles) was digested with NheI and HindIII to replace the coding sequence of eGFP by those from wt-αSyn and sp2-αSyn (obtained from the pcDNA3-CMV-hSyn-CMV-based plasmid).

To obtain the constructs encoding the N- and C-terminal ends of the fluorescent Venus protein (VN and VC), eGFP was excised from the pAAV-CMV-hSyn-CMV-eGFP-WPRE vector and replaced by the VN and VC coding sequences obtained from PCR using the primers:

VN forward, TAAT**GCTAGC**ATGGTGAGCAAGG, reverse, TAGC**AAGCTT**ATTACTGCTTGTCGGCGGTG; VC forward, TAGC**GCTAGC**ATGGCAAAGAACGGCATCAAGG, reverse, TAGC**AAGCTT**ATTACTTGTACAGCTCG (NheI and HindIII restriction sites are indicated bold). To generate the vectors encoding VN-wt-αSyn and sp2-αSyn-VC, a PCR product containing VN or VC sequences and generated with the primers:

VN-(wt-αSyn) forward, TAAT**GCTAGC**ATGGTGAGCAAGG, reverse, CCACCTCCGCCGCTTCCGCCACCATCGATCTGCTTGTCGGCGGTGAT (NheI restriction site is indicated bold and the DNA sequence of the linker IDGGGSGGGGSLK connecting VN and wt-αSyn is underlined); (sp2-αSyn)-VC forward, AAGAACGGCATCAAGG, reverse, CCTTGATGCCGTTCTTGGCTTCAGGTTCGTAG, was annealed with an overlapping PCR product of wt- or sp2-αSyn, respectively, created using the primers: (VN)-wt-αSyn forward, GGAAGCGGCGGAGGTGGAAGTCTTAAAGATGTATTCATGAAAGG, reverse, TCC**AAGCTT**TCATTAGGCTTCAGG (HindIII restriction site is indicated bold and the DNA sequence of the linker IDGGGSGGGGSLK connecting VN and wt-αSyn is underlined); sp2-αSyn-(VC) forward, TTA**GCTAGC**ATGGGAGTCAAAGTTCTGTTTGCCCTGATCTGCATCGC, reverse, CCTTGATGCCGTTCTTGGCTTCAGGTTCGTAG (NheI restriction site is indicated bold).

### Quantification of sp2-αSyn-VN/VC-wt-αSyn oligomers and aggregates released by secondary secretion

The amount of sp2-αSyn-VN/VC-wt-αSyn oligomers and aggregates released by secondary secretion was determined in fresh conditioned media from co-cultured cells. Around 10×E7 cells from each co-culture were harvested, seeded, and grown for 16 h at 37 °C. Then, cells were washed twice with pre-warmed media and fresh media was added. Collection of media of each culture was carried out every 10 min. Then the media was centrifuged at 1000×*g* for 30 min and finally concentrated 10-fold and used for fluorescence determination and Western blot.

### ThT staining

For ThT staining of cultured cells, cells were seeded onto a coverslip and 24 or 48 h after transfection were washed in PBS-Mg buffer (PBS supplemented with 10 mM Mg_2_Cl) and then fixed in 4% paraformaldehyde for 10 min. Fixed cells were then stained with a solution of 0.005% ThT for 10 min and then subjected to three washes of 5 min with 80% ethanol plus three washes with deionized water. Finally, the cells were mounted with the Immu-mount reagent (Thermo Scientific).

### Sequential extraction with detergents

Cos7 whole cell extracts (WCL) were homogenized in 100 µl of TBS+ buffer (50 mM Tris–HCl, 175 mM NaCl, 5 mM EDTA, and a protease inhibitor cocktail (Calbiochem, CA), pH 7.4) and centrifuged for 30 min at 120,000×*g*. Pellets were sequentially extracted in: (1) TBS+ containing 1% of Triton X-100; (2) TBS+ containing 1% of Sarkosyl; and (3) 8 M urea containing 5% SDS. Each extraction step was followed by centrifugation for 20 min at 120,000×*g*.

### Transfections

Transfection of plasmids (2 µg of total DNA) was carried out with Lipofectamine 2000 or Lipofectamine RNAiMAX (Invitrogen) transfection reagent, respectively, according to the manufacturer’s instructions. Unless otherwise indicated, cells were treated 48 h post-transfection and then extensively washed before harvest.

### Parallel reaction monitoring mass spectrometry

Four tryptic peptides of human α-Synuclein (2 + EGVVAAAEK, 2 + EGVLYVGSK, 2 + EQVTNVGGAVVTGVTAVAQK, and 2 + EGVVHGVATVAEK), were quantified by parallel reaction monitoring mass spectrometry (PRM) as surrogates for intracellular unmodified αSyn protein levels. The N-terminal acetylated tryptic peptide 2 + (Acetyl)-MDVFMK and the semi-tryptic sp2-peptide 2 + MGVKVLFALICIAVAEADVFMK were quantified similarly. For each target peptide, at least four PRM transitions of the y-series of the resulting fragment ions were used for the determination of peptide quantities. Dissolved samples were injected by an M-class UPLC system (Waters) operating in trap/elute mode. A Symmetry C18 trap column (5 µm, 180 µm × 20 mm (Waters)) and an HSS T3 C18 reverse-phase column (1.8 µm, 75 µm × 250 mm (Waters)) as separation column were used. The columns were equilibrated with 99% solvent A (0.1% formic acid (FA) in water) and 1% solvent B (0.1% FA in acetonitrile). Trapping of peptides was performed at 15 µl/min for 30 s and afterwards, the peptides were eluted using the following gradient: 1–40% B in 30 min and 40–98% B in 5 min. The flow rate was constant at 0.3 µl/min and the temperature was kept constant at 50 °C. High-accuracy mass spectra were acquired with a Q-Exactive HF mass spectrometer (Thermo Fisher Scientific) that was operated in PRM mode. Data were analyzed using Skyline (MacCoss, Version 3.7). Relative abundance of each peptide across different conditions is shown as the PRM transitions of each peptide visualized with Skyline. Results are expressed as counts per second of each PRM transition (at least four per peptide) over retention time.

### Expression and purification of recombinant wt-αSyn and sp2-αSyn

Recombinant wt-αSyn was produced as previously published^82^. Briefly, the pRK172 plasmid containing the wt-αSyn-coding sequence was transformed into *E. coli* BL21 Star (DE3) cells and then expressed as ^15^N-uniformly labeled protein as follows: a pre-culture of 200 ml was prepared with LB media and grown overnight at 37 °C in agitation (120–180 rpm). Then 20 ml of the preculture was added to each one of six flasks containing 2 L (1:100 dilution) of 2XLB media (20 g/L NaCl, 20 g/L tryptone, and 10 g/L yeast extract). These cultures were grown at 37 °C to OD_600_ = 1, the cells were then collected by centrifugation (4000×*g* for 15 min) and finally transferred into three flasks containing each 2 L of minimal media containing the isotopes. After 2 h IPTG (1 mM final concentration) was added to the cells, which were incubated overnight at 37 °C in agitation. Cells were harvested by centrifugation and stored as cell pellets at –80 °C until they were purified. For sp2-αSyn a different strategy was used due to the very low yield using the protocol above. The coding sequence of sp2-αSyn was cloned into the pRK172 vector using the restriction enzymes NdeI and HindIII. A thrombin recognition site and a 6XHis tag were added to the C-terminus of sp2-αSyn using the following primer 5’ CGATAAGCTTCATTCACTAATGATGATGATGATGGTGGCTGCCGCGCGGCACCAGGGCTTCAGGTTCGTAGTCTTG 3’. This construct was then transformed into BL21(DE3)pLysS *E. coli*. Expression was induced as with wt-αSyn and purification was done using a HisTrap FF column (GE Healthcare) on cleared cell lysates. Elution was achieved with 300 mM imidazole. Fractions containing recombinant sp2-αSyn-6XHis were pooled and dialyzed against 20 mM Tris–HCl pH 7.4. The protein solution was then lyophilized. Prior to NMR experiments, the protein was resuspended in PBS buffer and treated ON at 4 °C with thrombin (SIGMA) to remove the 6XHis tag following the manufacturer’s instructions. The protein solution was then passed through a HisTrap FF column to remove the 6XHis tag and then concentrated using a 3 kDa cut-off filter. For some NMR experiments, the 6XHis tag was not removed as the behavior of tagged and non-tagged sp2-αSyn remained unchanged.

### Fibrillization monitoring by thioflavin-T

Recombinant wt-αSyn and sp2-αSyn were resuspended in sterile PBS buffer pH 7.4 supplemented with 0.05% sodium azide. The samples (∼300 μl) were incubated in a final concentration of 250 μM at 37 °C for the indicated times in a thermomixer set at 700 rpm. The ThT binding assay was performed using a 25 μM ThT solution in 25 mM sodium phosphate, pH 6.0. Aliquots (30 μl) of the protein samples containing wt-αSyn and sp2-αSyn were diluted into the ThT buffer, and fluorescence emission was measured at 25 °C with an excitation wavelength of 440 nm. Fluorescence emission was recorded at 484 nm on a Cary Eclipse Fluorescence Spectrophotometer (Agilent).

### NMR spectroscopy

NMR experiments were performed on Bruker 700 and 900 MHz Avance III HD spectrometers equipped with a cryogenically cooled proton-optimized ^1^H[^13^C/^15^N] TCI probe. Specifically, 2D ^15^N–^1^H SOFAST HMQC spectra were acquired with a data size of 128 × 512 complex points for a sweep width (SW) of 28.0 ppm (^15^N) and 16.7 ppm (^1^H), 512 scans, 100 ms recycling delay (acquisition time ∼4 h). NMR spectra were processed with Topspin (Bruker) and Sparky (University of California, San Francisco). Visualization and data analysis were carried out in Sparky. All NMR experiments were performed in NMR buffer (PBS, pH 7.4) and at a temperature of 283 K if not otherwise indicated.

For in-cell NMR experiments, up to three plates of 90% confluent A2780 cells were used for one in-cell NMR experiment. Cells were grown in DMEM, pH 7.4 supplemented with 10% fetal calf serum, 4 mM l-glutamine, 100 U/ml penicillin, and 100 mg/ml streptomycin for 2 days, then harvested, pooled, centrifuged at 200×*g* for 3 min, and kept at 37 °C for 10 min as cell pellets. A lyophilized aliquot of ^15^N-labeled αSyn containing 2–5 mg of protein was re-suspended in 200 µl of sterile PBS pH 7.4 (GIBCO) and sonicated for 5 min in a bath sonicator. The αSyn sample was then mixed at a 1:1 ratio with electroporation buffer, and this mixture was then used to resuspend the cell pellets. Cells were kept for 5 min in the electroporation mixture containing αSyn and finally subjected to electroporation using a Neon electroporator (Invitrogen) following previous works protocols^82^. Immediately after electroporation, cells were washed twice with pre-warmed media, plated, and incubated at 37 °C for 4 h. After this recovery period, the cells were harvested by mild trypsinization, washed once with pre-warmed media, and once with PBS pH 7.4 containing 5% D_2_O. the cells were then collected in the NMR (Shigemi) and decanted by a soft centrifugation step of 300×*g* for 3 min.

### Production of viral AVV9 particles

To produce recombinant AAV9 viral stocks, Hek-293T cells (5.0 × 10^6^ cells per 10 cm plates) were co-transfected, in a 1:1 molar ratio, with the plasmid encoding eGFP or the αSyn variants (3 μg/plate) together with the helper/packaging plasmid pD1rs (10 μg/plate) expressing the AAV viral genes (rep gene from AAV serotype 9 and cap gene from AAV serotype 1) and the adenoviral genes required for AAV replication and encapsidation (Plasmid Factory, Heidelberg, Germany). Fifty hours post-transfection, the medium was discarded and the cells were harvested by low-speed centrifugation and resuspended in 50 mM Tris pH 8.5, EDTA 1 mM, NaCl 0.1 M. After three cycles of freezing/thawing, the lysate was clarified by 30 min centrifugation at 10,000×*g*, treated with benzonase (50 units/ml, Sigma) at 37 °C for 30 min, and centrifuged at 10,000×*g* for 30 min to eliminate the residual debris. The viral particles were further purified by an iodixanol gradient consisting of 15%, 25%, 40%, and 60% iodixanol. Viral particles were then concentrated until around 200 μl. Viral genomes (vg) were titrated using a quantitative polymerase chain reaction. Titers were 1.97 ± 0.36 × 10^12^ vg/ml for AAV9-eGFP, 1.43 ± 0.11 × 10^12^ vg/ml for AAV9-wt-αSyn, 2.33 ± 0.52 × 10^12^ vg/ml for AAV9-Sp2-αSyn.

### Animal experiments

Female mice were housed in a 12-h light/dark cycle with regular chow and water available ad libitum. All experiments were performed using 3–4-month-old mice (21–27 g body weight) on a pure C57BL/6J background. Experiments were carried out in strict accordance with the recommendations of the Guide for the Care and Use of Laboratory Animals of the National Research Council, USA (Guide for the Care and Use of Laboratory Animals. National Academies Press (2011). Available at: https://www.ncbi.nlm.nih.gov/books/NBK54050/ and all efforts were made to minimize suffering. All protocols received approval from the Institutional Animal Care and Use Committee of the IMBICE (ID 01-09-16).

### Stereotactic surgeries

Mice were randomized and assigned to one of the following experimental groups: AAV9–eGFP; AAV9-wt-αSyn; AAV9-Sp2-αSyn. All animals were injected at stereotactic coordinates used for the right mouse Substantia nigra with 1 μl of AAV that corresponds to viral dose 2E + 12 viral genomes (vg)/ml. All surgical procedures were performed using aseptic techniques and ketamine 100 mg/kg intraperitoneal [i.p.] and 10 mg/kg xylazine [i.p.] anesthesia. After confirming the absence of the pedal withdrawal reflex, the rodents were placed in a stereotactic head frame. The skull was exposed, by cutting along the midline, a small hole was drilled using a surgical drill and the needle was placed at the following coordinates from bregma: anteroposterior (AP), −0.3 mm; mediolateral (ML), −1.2 mm; and dorsoventral (DV), −4.5 mm or AP, +0.2; ML, −2.0; DV, −2.6. Injections will be performed using a 10 μl Hamilton syringe at a rate of 0.2 μl per min (1 μl total per site), the needle was left in place for an additional 5 min before being retracted. During the intervention mice were treated with subcutaneous injections of flunixin (2 mg/kg) in order to alleviate postoperative complications and the eyes were kept moisturized. The skin was sutured and anesthesia was reversed with an i.p. injection of yohimbine (2 mg/kg). After the surgery, the animal was placed on a heating pad until it began to recover before being returned to its cage. All operated mice were maintained under highly hygienic conditions and were monitored regularly following recovery from surgery.

### Histology of mouse tissue

At different time points after injection (2 w.p.i. or 4 m.p.i.), animals were anesthetized and transcardially perfused with phosphate-buffered saline (PBS) followed by ice-cold formalin. The brain was removed, post-fixed in the same fixative for 4 h at 4 °C, immersed in 20% sucrose in PBS, pH 7.0 at 4 °C, and cut coronally at 30 µm into four equal series on a sliding cryostat. Immunohistochemistry was performed on free-floating sections using specific antibodies against alpha-synuclein or tyrosine hydroxylase. Briefly, sections were pretreated with 1% H_2_O_2_, treated with a blocking solution, and incubated with anti-alpha synuclein antibody (anti-alpha-synuclein antibody [LB 509], Abcam, ab27766 1∶3000) or anti-TH antibody (AB152, Chemicon, 1:1000) ON at 4 °C. Then, sections were treated with biotinylated anti-mouse or anti-rabbit antibody for 1 h, and with Vectastain Elite ABC kit (Vector Laboratories, Burlingame, CA) for 1 h, according to manufacturer’s protocols. Then, the visible signal was developed with a 3-3′-diaminobenzidine (DAB) solution. For fluorescent double staining, sections were rinsed three times in PBS, treated with a blocking solution, and then incubated overnight in PBS-0.3% triton X-100, 3% goat serum, and the corresponding antibodies (see STAR table). After three rinses in PBS-0.04% triton X-100, the sections were incubated in the dark for 4 h in fluorochrome-conjugated secondary antibodies. After being rinsed in PBS, the sections were incubated for 30 min with DAPI, washed again, and coverslipped with mounting medium (Immu-Mount, Thermo Scientific). Fluorescent staining was visualized with a confocal Zeiss Spinning Disk microscope.

### Behavioral testing: Rotarod

The rotarod test was used to assess motor coordination and balance in the mice expressing eGFP or each αSyn variant after 4 months of injection. First, the animals were trained to reach stable performance. The training consisted of three trials of 60 s with 10 min inter-trial intervals at a fixed speed of 4 RPM. After this initial training, the mice were subjected to the test, and the latency to fall was recorded. The test phase consisted of three trials separated by 15 min inter-trial intervals where the rotarod was set to accelerate from 4 to 14 (slow acceleration) or 4–40 RPM in 300 s, and animals from the same cage were placed in separate lanes on the rod initially rotating at 4 RPM. The trials began when acceleration was started and finished when the animal fell off the rod.

### Open field test

The open field test was used to assess locomotor behavior in the mice expressing eGFP or each αSyn variant after 4 months of injection. Single-housed mice were transferred inside their home cage to the testing room 10 min before the trial to minimize the effects of stress on behavior during testing. Each mouse was then placed in a square-bottomed, white acrylic test arena (30 × 30 × 22 cm) inside a mechanically ventilated and acoustically isolated closed monitoring box (55 × 35 × 90 cm) equipped with an overhead camera and controlled LED illumination, and its activity was recorded for 20 min at 30 frames per second with a linked computer. All experiments were carried out between 9:00 and 15:00. The arena was cleaned with 70% ethanol between trials. Videos were imported into Fiji, re-sampled at 8 frames per second, and processed using custom-made macros to extract the position of the mouse within the arena, and to calculate the distance traveled and time spent in each zone. A square region located in the middle of the arena and with half its area was considered to compute the time and distance associated with the center. For the purpose of analysis, consecutive 5-min-long bins were used.

### Immunofluorescence analyses of cultured cells

For immunofluorescence analysis of cultured cells, cells were seeded onto a coverslip and, after transfection and/or treatment, were fixed in 4% paraformaldehyde. Fixed cells were washed three times with Mg–PBS buffer (10 mM MgCl in phosphate buffer pH 7.4) and permeabilized with Mg–PBS buffer supplemented with 0.5% Tritton X-100. Coverslips were washed five times and incubated with a blocking solution (3% of BSA fraction V in Mg–PBS buffer) for 10 min. Primary (dilution 1:3000, 2 h incubation at room temperature), as well as Alexa-fluo-conjugated secondary (dilution 1:5000, 1 h incubation at room temperature in the dark) antibodies, were prepared in blocking solution. DAPI staining was carried out in Mg–PBS buffer for 5 min in the dark.

### Western blot

WB was carried out following standard procedures. All blots were processed in parallel and derived from the same experiments.

### Statistics

For in vitro studies we used unpaired two-tailed Student’s *t*-test when only two groups were compared, ANOVA with a Dunnett’s test for multigroup comparisons where every group/treatment is compared with a single control, one-way ANOVA followed by a Fisher test for multi-comparisons within experiments of small sample sizes, and one-way ANOVA followed by a Tukey’s test for all possible pairwise comparisons. *P*-value of 0.05 or lower was considered statistically significant. Additional information is provided in figure legends. For studies in mice, we used paired two-tailed Student’s *t*-test for experiments where only two groups of the same animal (not independent) are compared. Differences among means were assessed by one-way ANOVA with Dunnett’s post hoc test. The null hypothesis was rejected at the 0.05 level. All results are expressed as mean ± SEM. Statistical analyses were performed with Statistica v7 (StatSoft) and GraphPad Prism.

### Supplementary information

Supplemental Figures

## Data Availability

All data are available in the main text or the supplementary materials. All the plasmids generated in this work will be available through materials transfer agreements.
