# Supplementary material for: Cell-to-cell transmitted alpha-synuclein recapitulates experimental Parkinson’s disease
Source: NPJ Parkinsons Dis. 2024 Jan 6;10:10. doi: 10.1038/s41531-023-00618-6 (PMC10771530; doi:10.1038/s41531-023-00618-6)

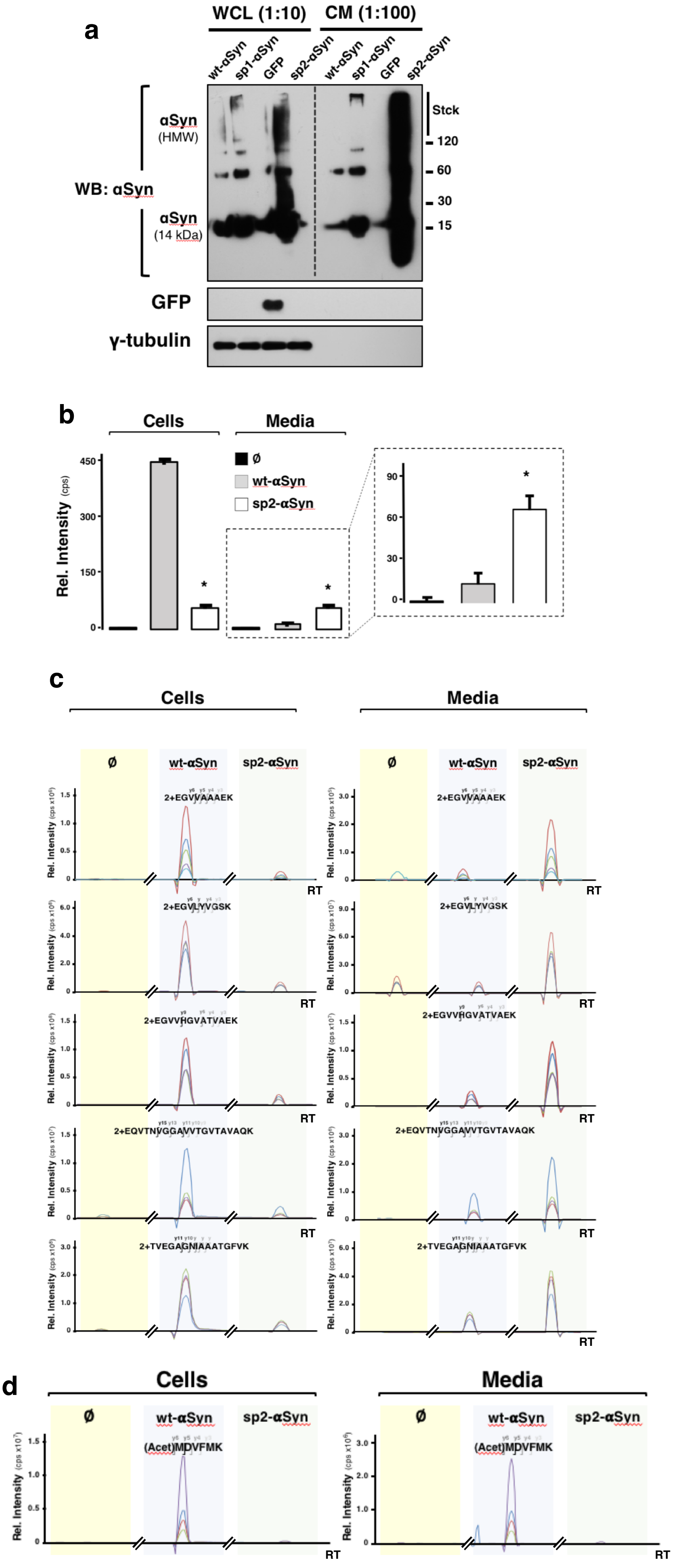
a
WCL (1:10)
CM (1:100)
wt-αSyn
sp1-αSyn
GFP
sp2-αSyn
αSyn (HMW)
WB: αSyn
αSyn (14 kDa)
Stck
120
60
30
15
GFP
γ-tubulin
b
Cells
Media
Rel. Intensity (cps)
Ø
wt-αSyn
sp2-αSyn
c
Cells
Media
2+EGVVAAAEK
2+EGVLYVGSK
2+EGVVHGVATVAEK
2+EQVTNVGGAVVTGVTAVAQK
2+TVEGAGNIAAATGFVK
RT
d
Cells
Media
(Acet)MDVFMK
RT

e

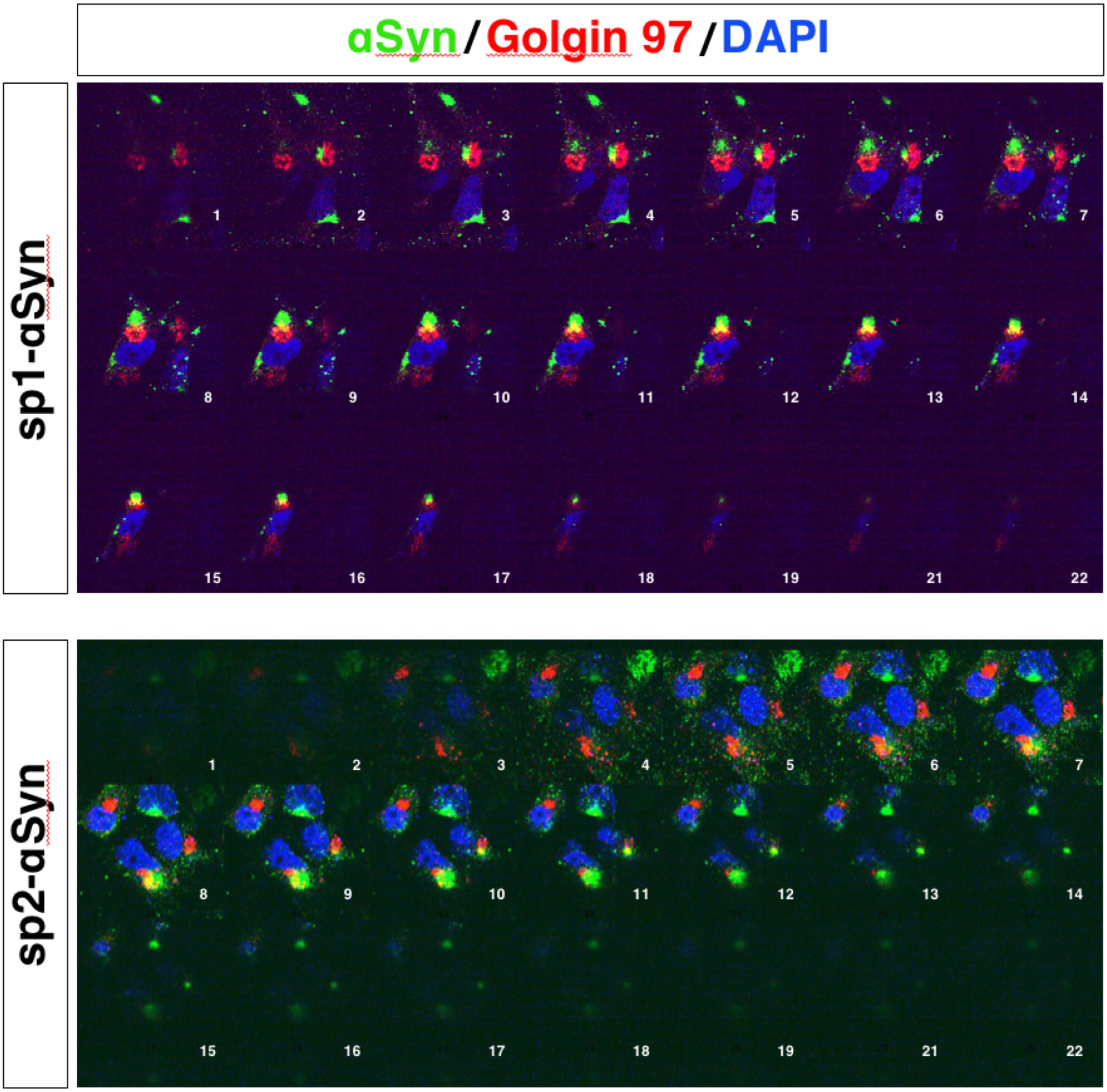
aSyn / Golgin 97 / DAPI
sp1-aSyn
1
2
3
4
5
6
7
8
9
10
11
12
13
14
15
16
17
18
19
21
22
sp2-aSyn
1
2
3
4
5
6
7
8
9
10
11
12
13
14
15
16
17
18
19
21
22

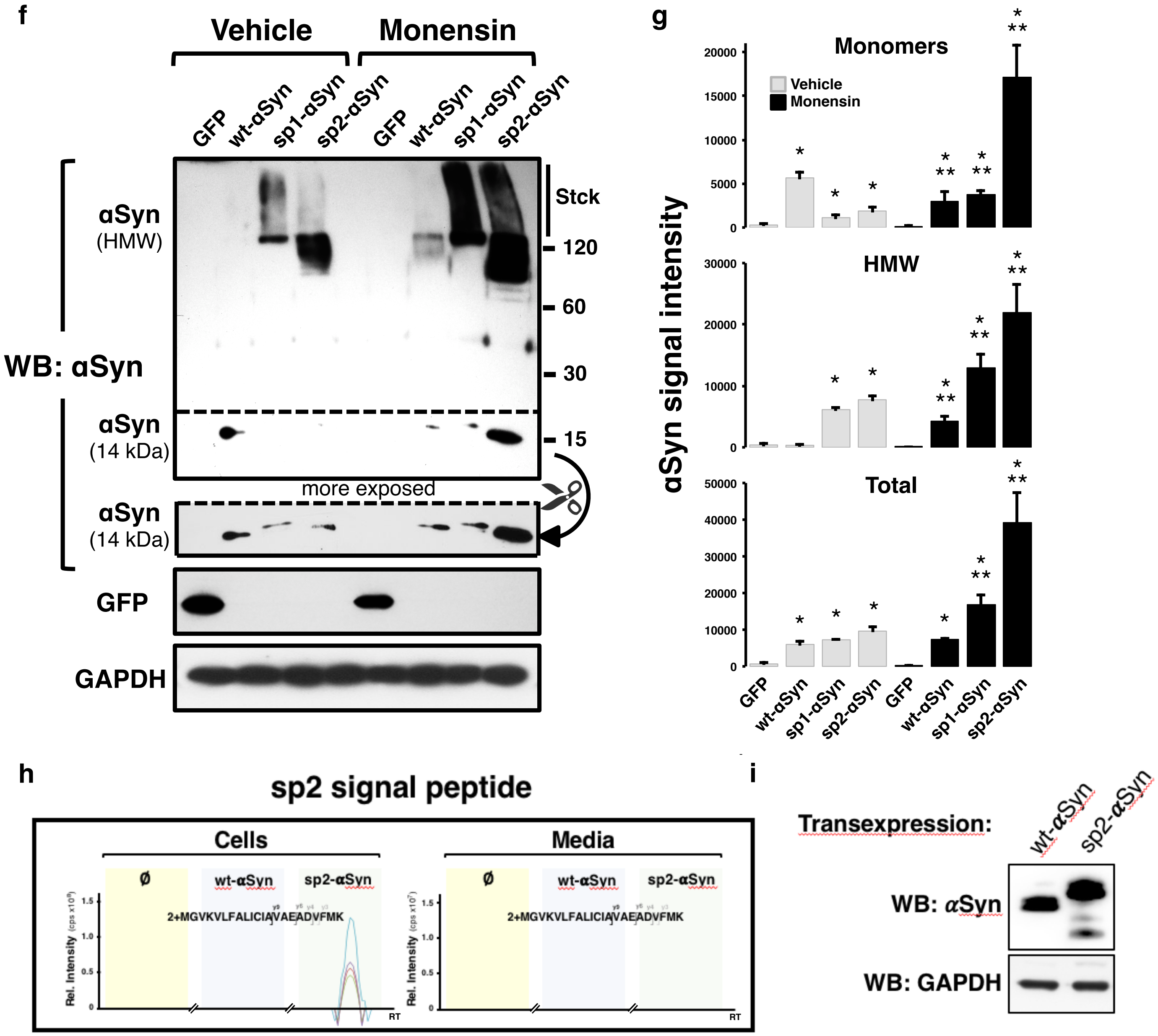
f
Vehicle
Monensin
GFP
wt-αSyn
sp1-αSyn
sp2-αSyn
GFP
wt-αSyn
sp1-αSyn
sp2-αSyn
αSyn
(HMW)
Stck
120
60
30
15
WB: αSyn
αSyn
(14 kDa)
more exposed
αSyn
(14 kDa)
GFP
GAPDH
g
αSyn signal intensity
Monomers
Vehicle
Monensin
HMW
Total
GFP
wt-αSyn
sp1-αSyn
sp2-αSyn
GFP
wt-αSyn
sp1-αSyn
sp2-αSyn
h
sp2 signal peptide
Cells
Media
Ø
wt-αSyn
sp2-αSyn
2+MGVKVLFALICIAVAEADVFMK
Rel. Intensity
RT
i
Transexpression:
wt-αSyn
sp2-αSyn
WB: αSyn
WB: GAPDH

j

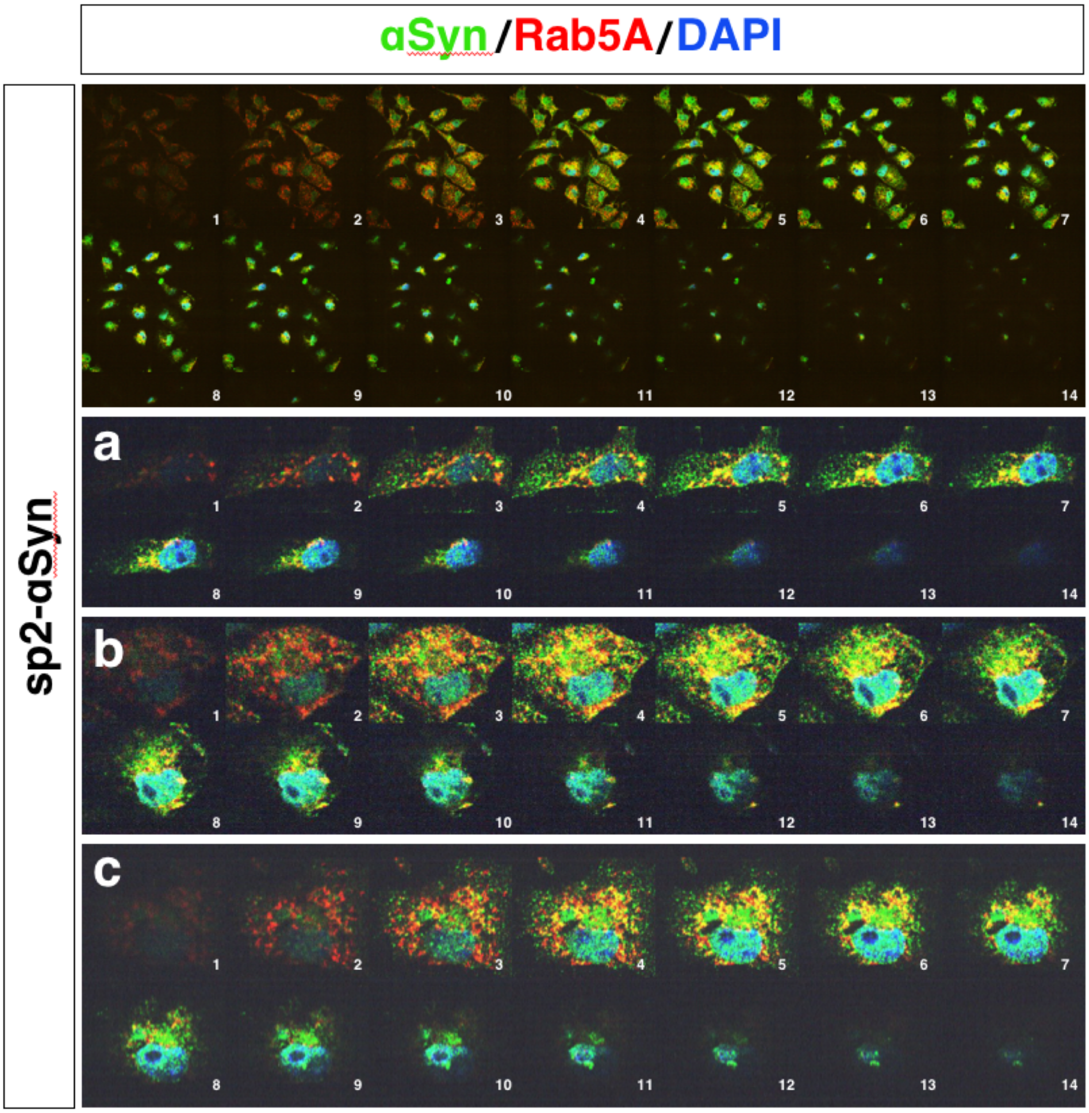

Supplementary Figure 1. sp-tagged αSyn are secreted and internalized. (**a**) Representative WB analyses of whole cell lysates (WCL, 1:10 from the total lysate) and conditioned media (CM, 1:100 from the total CM) of human neuroblastoma SH-SY5Y cells expressing GFP or wt-, sp1- or sp2-αSyn. Stck, stacking gel. (**b**) Quantification of the PRM traces of cell lysates ("Cells") and conditioned media ("Media") of cells transfected with an empty vector (Ø) or expression vectors for wt-αSyn or sp2-αSyn. The PRM traces are shown in (**c**) where the tryptic peptides targeted for PRM experiments are also indicated. Bar plots show averaged counts per seconds (cps) of PRM traces corresponding to the indicated tryptic peptides. Data is shown as the media ± SD. * $p < 0.05$ compared to empty vector (one-way ANOVA followed by the post hoc Tukey's test n=4). (**d**) PRM traces of cell lysates ("Cells") and conditioned media ("Media") of cells transfected with an empty vector (Ø) or expression vectors for wt-αSyn or sp2-αSyn. The acetylated αSyn N-terminus tryptic peptide (sequence: MDVFMK) was targeted for PRM experiments. (**e**) Z-stack images of selected panels of figure 1c. (**f**) Representative WB of WCL of Cos7 cells expressing GFP or wt-, sp1- or sp2-αSyn and treated with vehicle or monensin for 16 h. (**g**) Quantification of the signal intensity of the αSyn immunoblots of (**f**). Two areas of the gel were quantified separately, the lower part containing αSyn monomers and the upper part containing the HMW species. Total signal intensity was calculated as the sum of monomers and HMW. Data is shown as the media ± SD. * and **, $p < 0.05$ respect GFP and monensin, respectively (one-way ANOVA followed by the post hoc Tukey's test, n=3). (**h**) PRM traces of cell lysates ("Cells") and conditioned media ("Media") of cells transfected with an empty vector (Ø) or expression vectors for wt-αSyn or sp2-αSyn. The sp2 semi-tryptic peptide was targeted for PRM experiments. Cps, counts per second. RT, retention time. (**i**) Western blot analyses of whole cell lysates obtained from mammalian cells transexpressed with recombinant wt-αSyn and sp2-αSyn. Anti-αSyn antibodies were used to detect the transexpressed proteins whereas an anti-GAPDH antibody was used as loading control. (**j**) Z-stack images of selected panels of figure 1f.

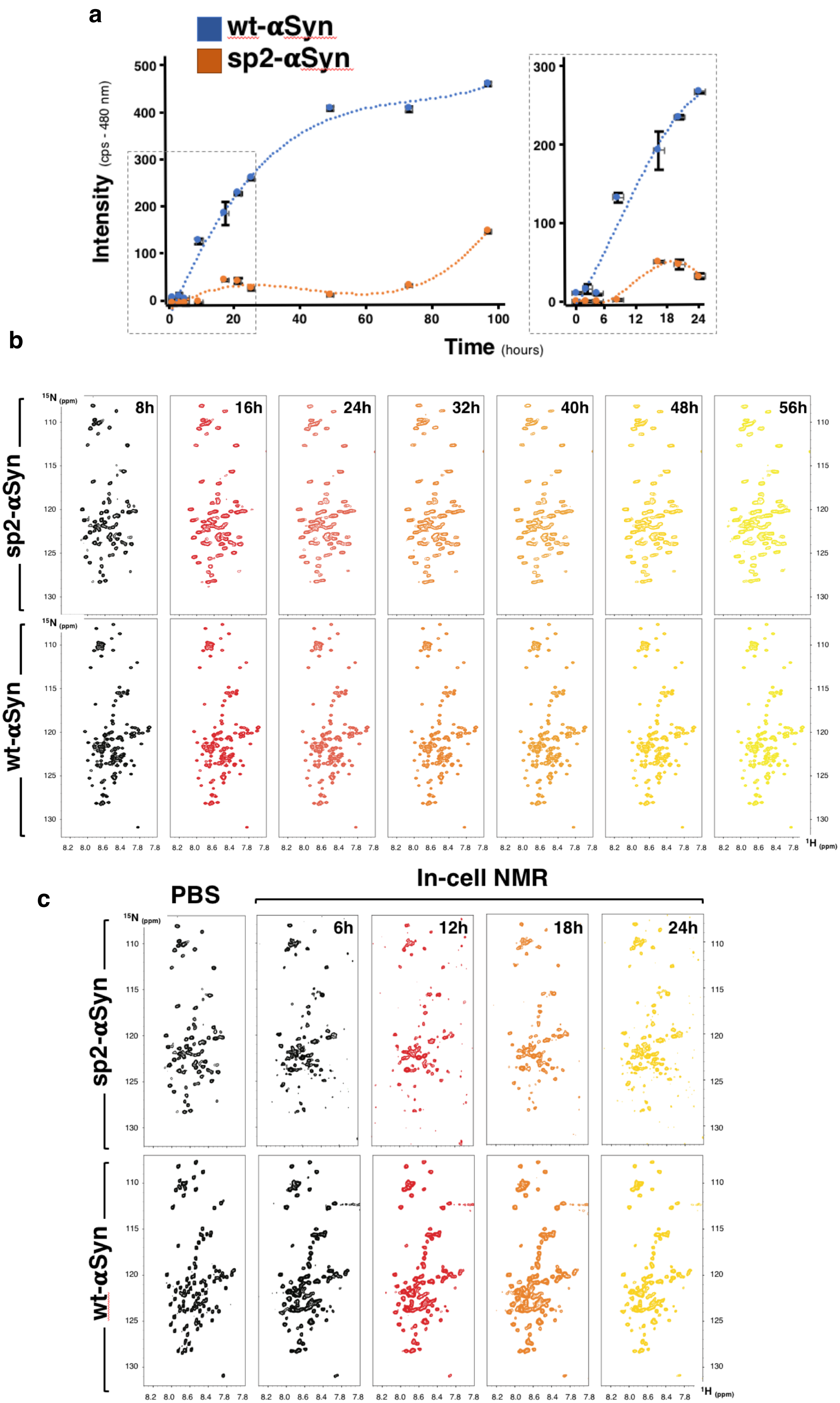

a
wt-αSyn
sp2-αSyn
Intensity (cps - 480 nm)
Time (hours)
b
sp2-αSyn
wt-αSyn
8h
16h
24h
32h
40h
48h
56h
^{15}N (ppm)
^{1}H (ppm)
c
PBS
In-cell NMR
6h
12h
18h
24h

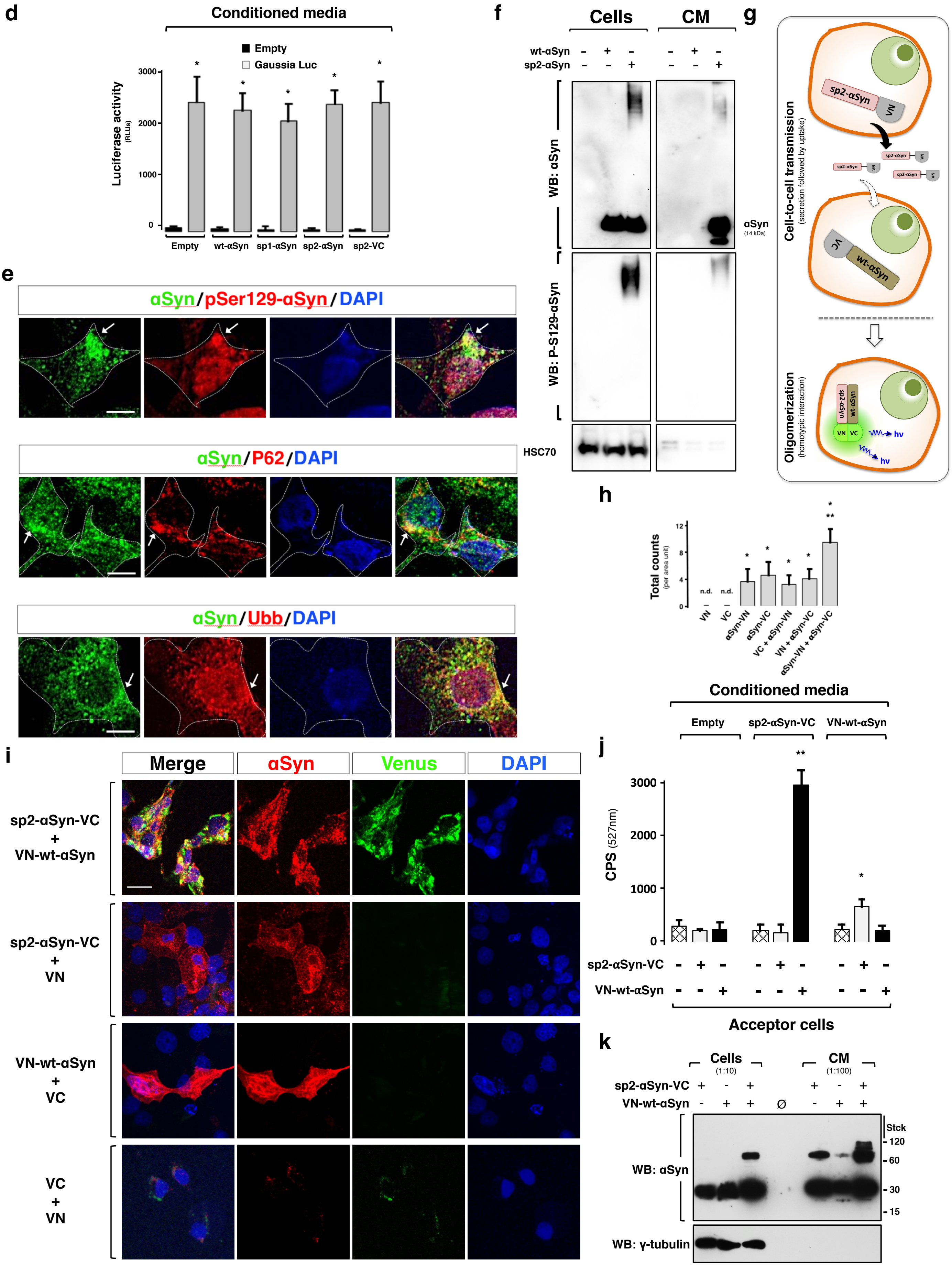
d
Conditioned media
Empty
Gaussia Luc
Luciferase activity (RLUs)
3000
2000
1000
0
Empty
wt-αSyn
sp1-αSyn
sp2-αSyn
sp2-VC
e
αSyn/pSer129-αSyn/DAPI
αSyn/P62/DAPI
αSyn/Ubb/DAPI
f
Cells
CM
wt-αSyn
sp2-αSyn
WB: αSyn
WB: P-S129-αSyn
αSyn (14 kDa)
HSC70
g
sp2-αSyn
VN
VC
wt-αSyn
Cell-to-cell transmission
(secretion followed by uptake)
Oligomerization
(homotypic interaction)
hν
h
Total counts (per area unit)
12
8
4
0
n.d.
VN
VC
αSyn-VN
αSyn-VC
VC + αSyn-VN
VN + αSyn-VC
αSyn-VN + αSyn-VC
i
Merge
αSyn
Venus
DAPI
sp2-αSyn-VC + VN-wt-αSyn
sp2-αSyn-VC + VN
VN-wt-αSyn + VC
VC + VN
j
Conditioned media
Empty
sp2-αSyn-VC
VN-wt-αSyn
CPS (527nm)
3000
2000
1000
0
sp2-αSyn-VC
VN-wt-αSyn
Acceptor cells
k
Cells (1:10)
CM (1:100)
sp2-αSyn-VC
VN-wt-αSyn
Ø
WB: αSyn
Stck
120
60
30
15
WB: γ-tubulin

Supplementary Figure 2. sp2-αSyn is toxic, amyloidogenic and cell-to-cell transmitted.

(**a**) Thioflavin-T fluorescence of recombinant wt-αSyn (blue) and sp2-αSyn (orange) incubated in PBS buffer at 37 °C in agitation for the indicated times. On the right a magnification of the 0-24 h is shown. (**b**) Two dimensional [^{15}N,^{1}H]-Heteronuclear Multiple Quantum Coherence (HMQC) NMR spectra of ^{15}N-labeled wt- and sp2-αSyn incubated in PBS buffer for the indicated times. NMR measurements were carried out at 10 °C. (**c**) Two dimensional [^{15}N,^{1}H]-HMQC NMR spectra of ^{15}N-labeled wt- and sp2-αSyn transexpressed in mammalian cells and kept in the NMR tube for the indicated times ("in cell-NMR"). The reference spectra correspond to the same proteins in buffer (first black spectra) is shown ("PBS"). NMR measurements were carried out at 10 °C. (**d**) Bar graph showing the luciferase activity recovered in CM of cells co-expressing the Gaussia luciferase and wt-, sp1-, sp2-αSyn or the control secreted protein sp2-VC. Data is shown as the media ± SD. * $p < 0.005$ (one-way ANOVA followed by the *post hoc* Tukey's test, n=5). (**e**) Cos7 cells expressing sp2-αSyn for 48 h were fixed and subjected to confocal microscopy analyses to visualize αSyn (green), αSyn phosphorylated at serine 129, P62 and ubiquitin (all them in red). DAPI (blue) was used for nuclei staining. Scale bar, 10 μm. (**f**) Western blot analyses of cell lysates ("Cells") and conditioned media ("CM") of cells transfected with an empty vector (-) or expression vectors for wt-αSyn or sp2-αSyn. Antibodies against αSyn and αSyn phosphorylated at serine 129 were used to detect the modified forms of this protein, whereas an anti-HSC70 antibody were used as loading control. (**g**) Schematic representation of the bimolecular fluorescence complementation (BiFC) approach used to study sp2-αSyn cell-to-cell propagation, seeded oligomerization and secondary secretion. Note that for visualization purposes the sp2 peptide was not removed from the secreted sp2-αSyn. (**h**) Cos7 cells expressing wt-αSyn-VC, VN-wt-αSyn or the control proteins VN or VC were grown alone or co-incubated as indicated. After 48 h the cells were fixed and subjected to confocal microscopy analyses to quantify cells containing the reconstituted Venus protein. Very few fluorescent cells (counts) were observed in wt-αSyn-VC and VN-wt-αSyn-expressing cells even in absence of the missing complementary GFP half (bars 3 and 4). Co-cultures of cells expressing wt-αSyn-VC and VN-wt-αSyn (bar 7) yielded few fluorescent cells which were more abundant compared to cells expressing these proteins alone. Data is shown as the media ± SD. * $p < 0.05$ compared to VN and VC. ** $p < 0.05$ compared to all other conditions. (**i**) Cos7 cells expressing sp2-αSyn-VC, VN-wt-αSyn or the control protein VC were co-incubated with cells expressing VN-wt-αSyn, VN or VC. After 48 h the cells were fixed and subjected to confocal microscopy analyses to visualize the reconstituted Venus protein. Scale bar, 10 μm. (**j**) Quantification of luciferase activity in acceptor cells expressing sp2-αSyn-VC (grey bars) or VN-wt-αSyn (black bars). Acceptor cells were treated with conditioned media containing VC-sp2-αSyn or VN-wt-αSyn. Data is shown as the media ± SD. * $p < 0.05$ compared to Empty. ** $p < 0.01$ compared to cells lacking αSyn (squared bars). Unpaired, two tails distribution Student's *t* test, (n=5). (**k**) HeLa cell clones expressing VN-wt-αSyn or sp2-αSyn-VC were co-cultured as indicated. 24 h later the CM was collected and subjected to WB together with the cell lysates (Cells). Ø, empty lane

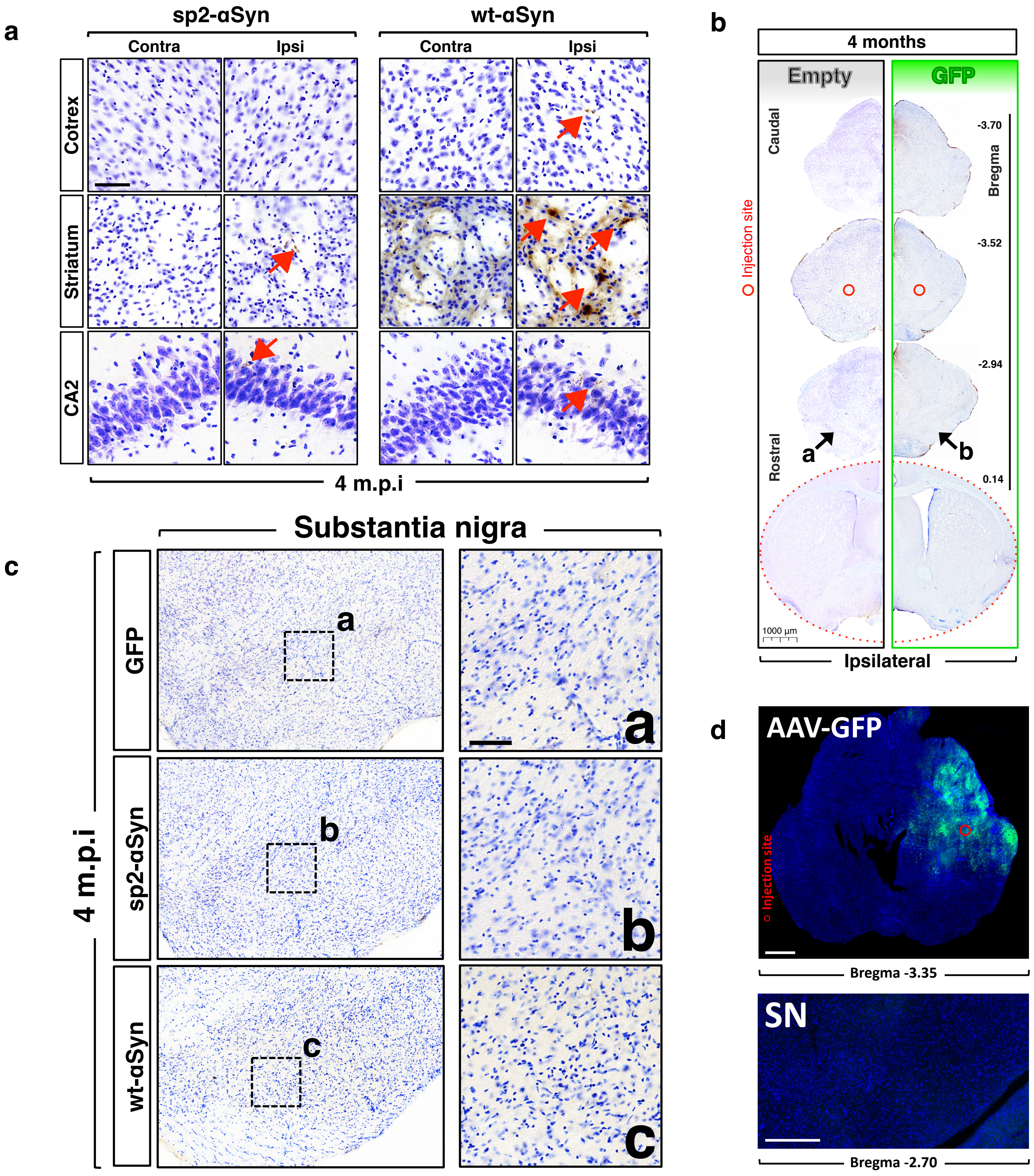

Supplementary Figure 3. **Neuron-derived extracellular αSyn triggers a robust LB-like pathology in the substantia nigra.** (**a**) Representative IHC images of coronal sections of cortex, striatum and the CA2 region of the hippocampus (CA2) of wild type mice transduced with AAV-sp2-αSyn or AAV-wt-αSyn for 4 months. Both contralateral (Contra) and ipsilateral (Ipsi) hemispheres are shown. Arrows, αSyn-positive inclusions found in neuronal bodies and fibers. Scale bar, 100 µm. (**b**) Representative IHC images of consecutive coronal sections of brains of wild type mice (n=6) transduced with an empty AAV9 (grey, Empty) or an AAV9 encoding GFP (green, GFP). Animals were sacrificed at 4 months post injection. Approximate rostrocaudal coordinates taking the bregma as reference are indicated on the right. Only ipsilateral hemispheres are shown. Arrows, substantia nigra. (**c**) Control IHC images of substantia nigra of wild type mice transduced with AAV-GFP, AAV-sp2- or AAV-wt-αSyn in for 4 months. Primary antibody was omitted in these control experiments. Scale bar, 100 µm. (**d**) Representative fluorescence images of mice expressing eGFP for 4 months in the DMN. The area where the virus was administrated and the substantia nigra are shown. Nuclei were stained with DAPI. Scale bar, 100 µm.

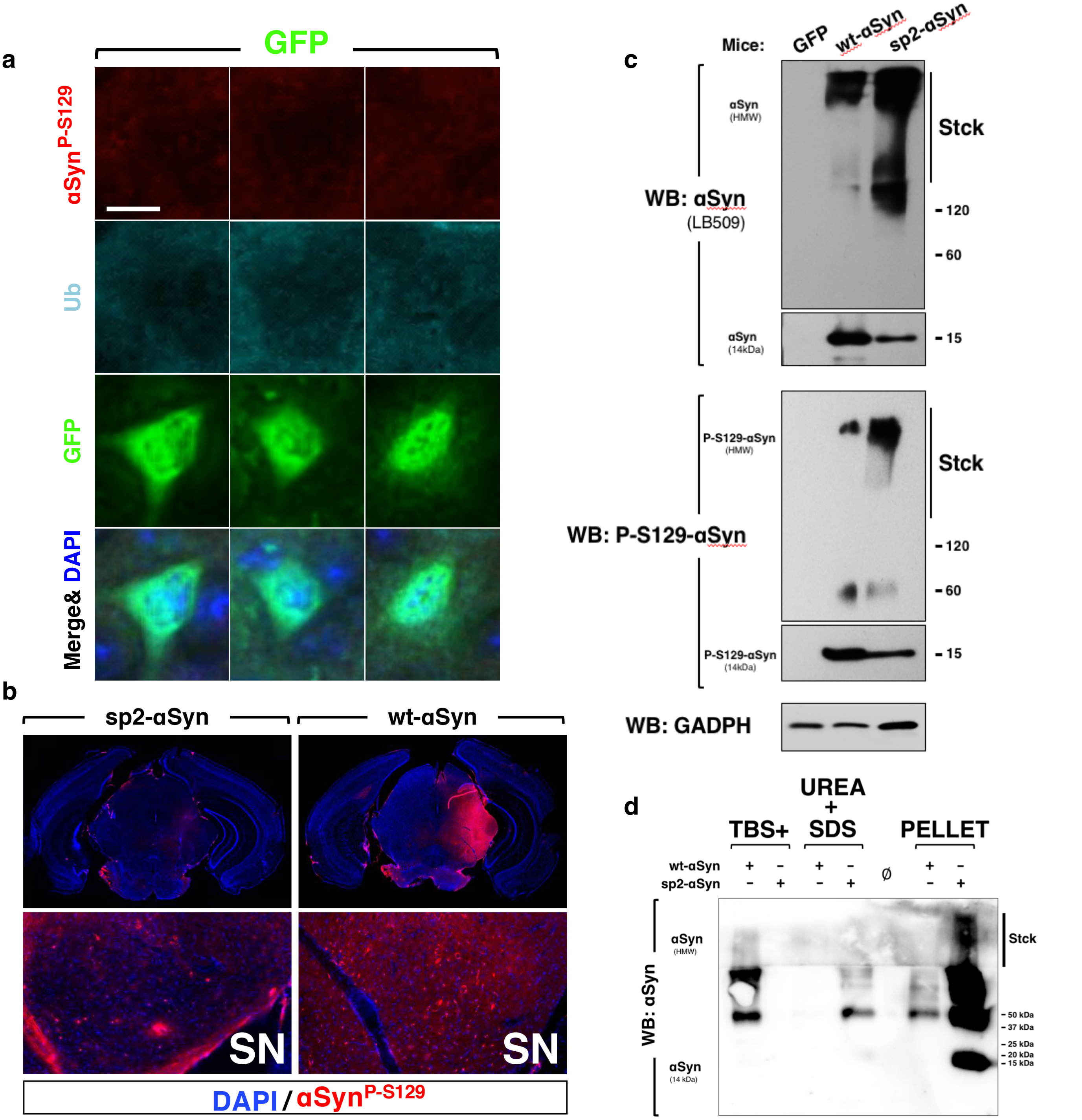

Supplementary Figure 4. Lewy body-like pathology elicited by sp2- and wt-αSyn. (**a**) Representative double immunofluorescence images of mice expressing GFP for 4 months. Immunostaining was carried out with antibodies specific for ubiquitin (Ub) and αSyn phosphorylated at serine 129. Nuclei were stained with DAPI. Scale bar, 10 µm. (**b**) Lower magnification images showing immunofluorescence analyses of brains expressing sp2- and wt-αSyn for 4 months. Immunostaining was carried out with an antibody that recognizes αSyn phosphorylated at serine 129. a higher magnification showing the substantia nigra is shown in the bottom. Nuclei were stained with DAPI. (**c**) Representative WB of brain tissue enriched of midbrain from mice transduced with AAV-GFP, AAV-wt- or AAV-sp2-αSyn. An anti-αSyn antibody (clone LB509) that recognizes human but not mouse αSyn was used for the immunoblot. Note that in this experiment we used four times more material from the sp2-αSyn mice due to lesser overall amount of αSyn compared to wt-αSyn-expressing mice (see GAPDH). (**d**) Representative WB of brain tissue enriched of midbrain from mice transduced with AAV-wt- or AAV-sp2-αSyn and subjected to sequential extraction with the indicated buffers. The LB509 anti-αSyn antibody was used. Stck, stacking gel. Four times more material from the sp2-αSyn mice was used due to lesser overall amount of αSyn compared to wt-αSyn-expressing mice.

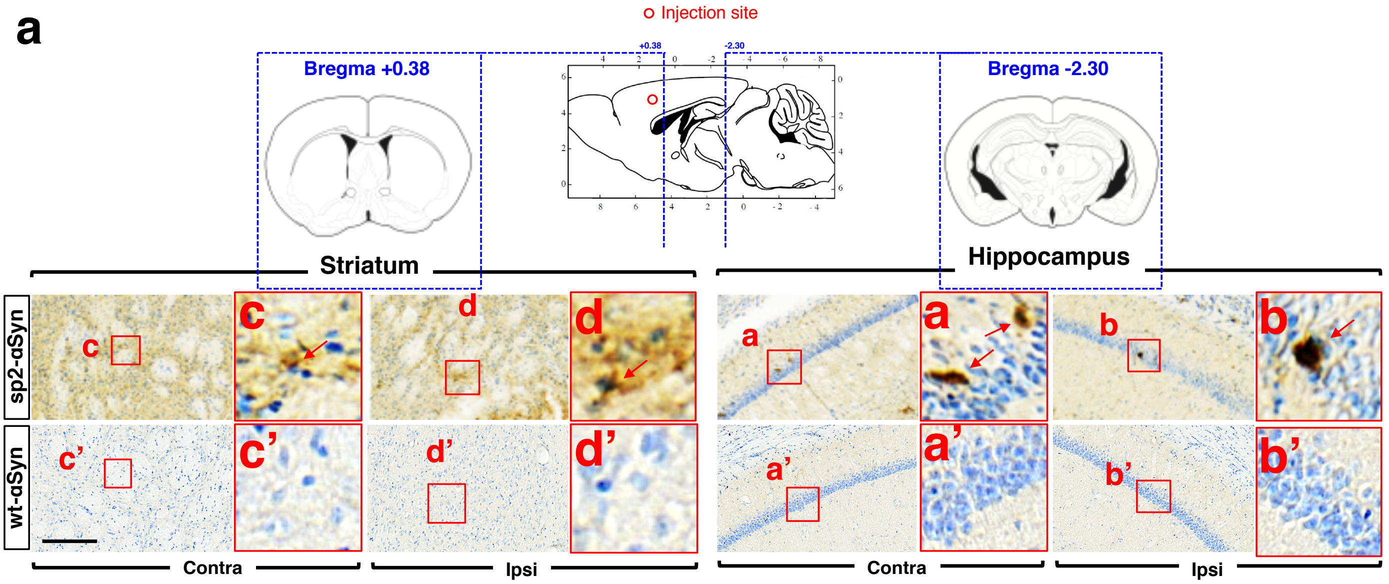

Supplementary Figure 5. sp2-αSyn spreading in the mammalian brain. (**a**) Representative IHC images of coronal brain sections of hippocampus and striatum of wild type mice that received a single injection of an AAV9 encoding sp2- or wt-αSyn in the cortex. Animals were sacrificed at 4 months post-surgery. In the upper part a schematic representation of the anatomical structures analyzed that includes approximate rostro-caudal coordinates with the bregma as reference. Contralateral (Contra) and ipsilateral (Ipsi) hemispheres are shown. Scale bar: 200 µm.

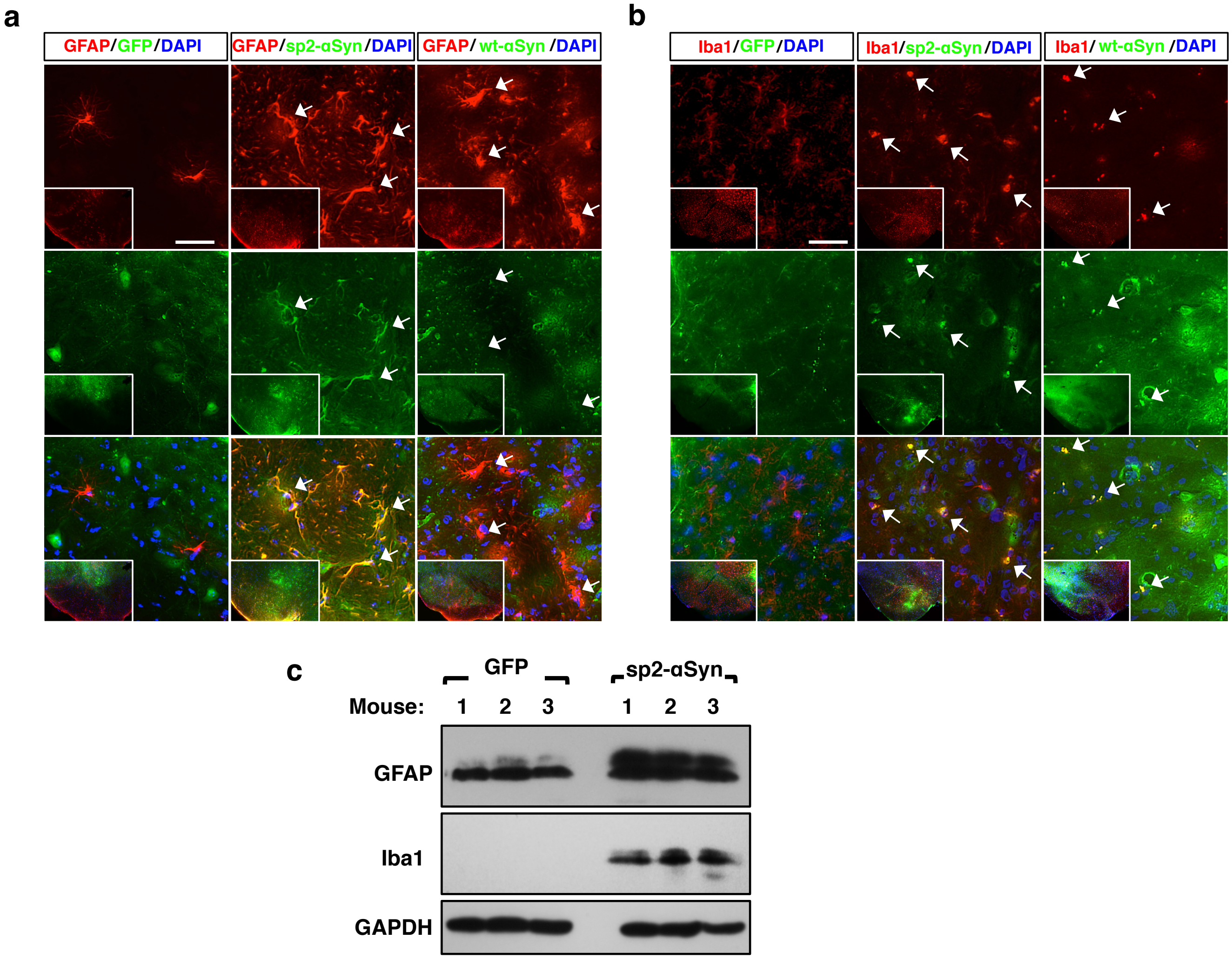

Supplementary Figure 6. sp2-αSyn triggers neuronal death and neuroimmflamation. (**a** and **b**) Representative immunofluorescence images of the substantia nigra of mice transduced with GFP, sp2- or wt-αSyn AAVs for 4 months. Human αSyn (green) was co-immunostained with the marker for astrocytes GFAP (**a**) or microglia Iba1 (**b**). Arrows indicate co-localization between the glial marker and αSyn in astrocytes or microglia. DAPI was used for nuclear staining. Scale bar: 100 µm. (**C**) Representative WB of brain tissue from mice transduced with AAV-GFP (n=3) or AAV-sp2-αSyn (n=3) for 4 months.

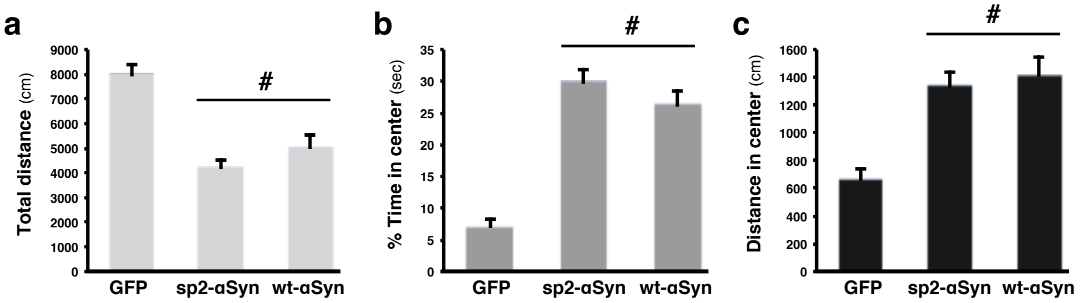

Supplementary Figure 7. Locomotor deficits elicited by sp2-αSyn. (**a-c**) Plots of the total distance travelled (**a**), time (in percentage) that the animals occupied the center (**b**) or distance travelled in the center (**c**) derived from the open field test (20 minutes sessions). Mice expressing GFP (n=6), sp2- (n=6) or wt-αSyn (n=5) for 4 months were used in this study. Data is shown as the media ± SD. # $p < 0.05$ (one-way ANOVA followed by the *post hoc* Dunnett's test).

Western blotting, uncropped blots

Figure 1b

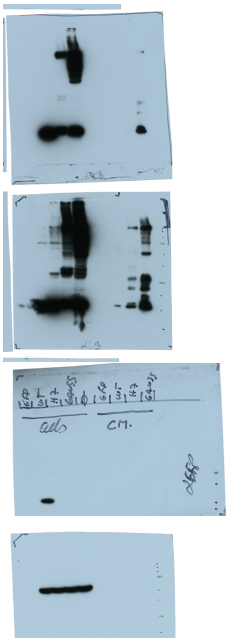

Figure 1d

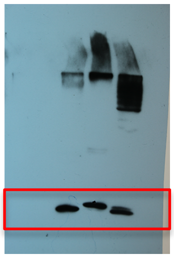

Figure 1e

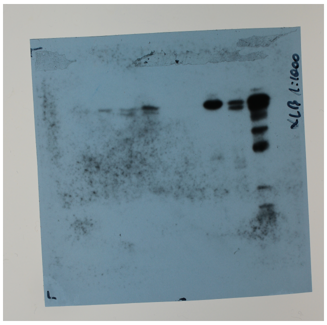

Supplem. Figure 1a

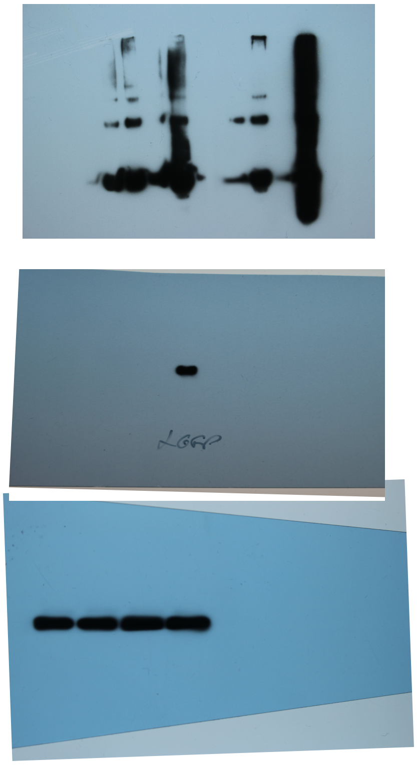

Supplem. Figure 1i

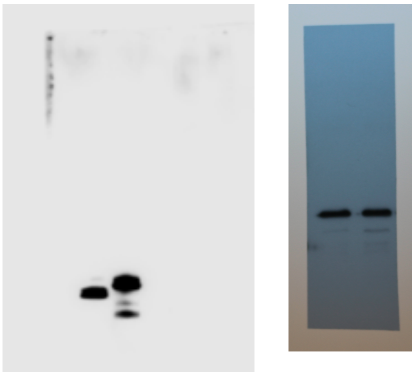

Supplem. Figure 1f

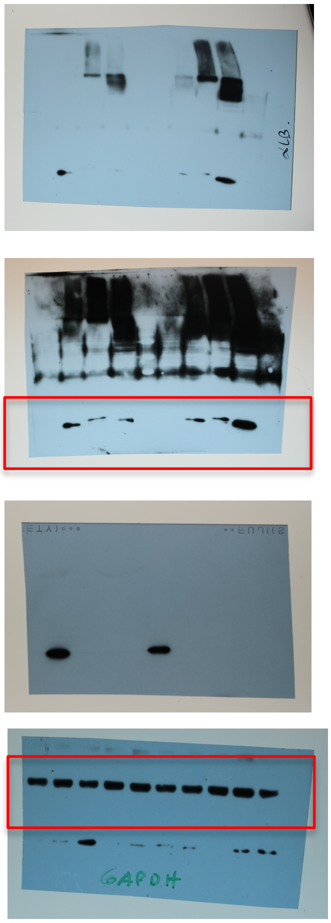

Figure 2d

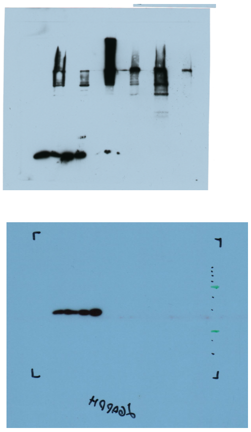

Supplem. Figure 2f

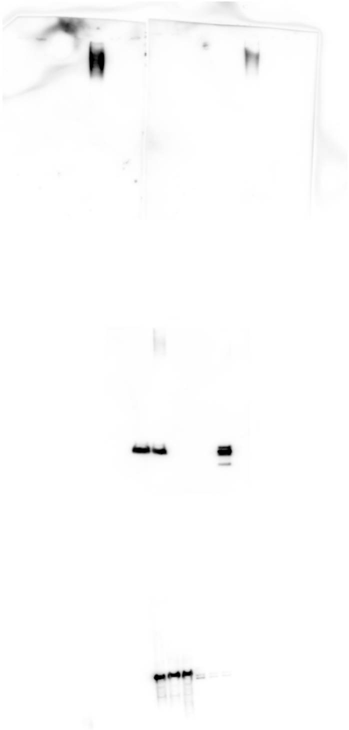

Supplem. Figure 2k

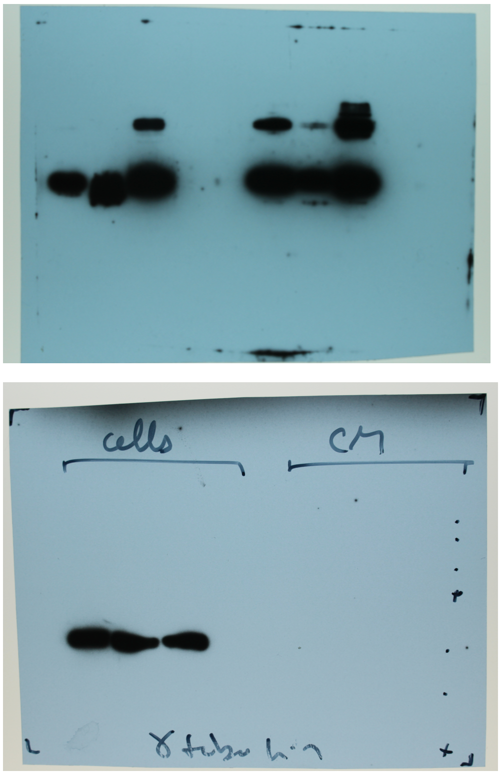

Supplem. Figure 4c

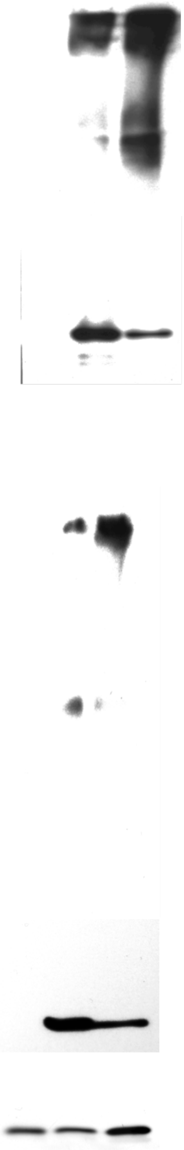

Supplem. Figure 4d

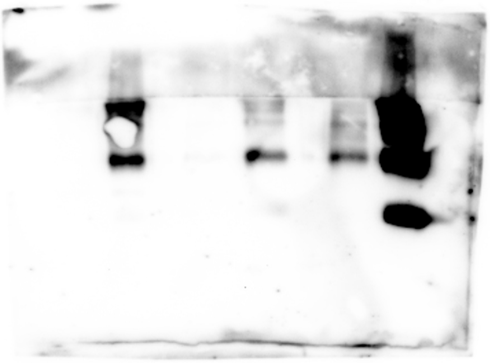

Supplem. Figure 6c

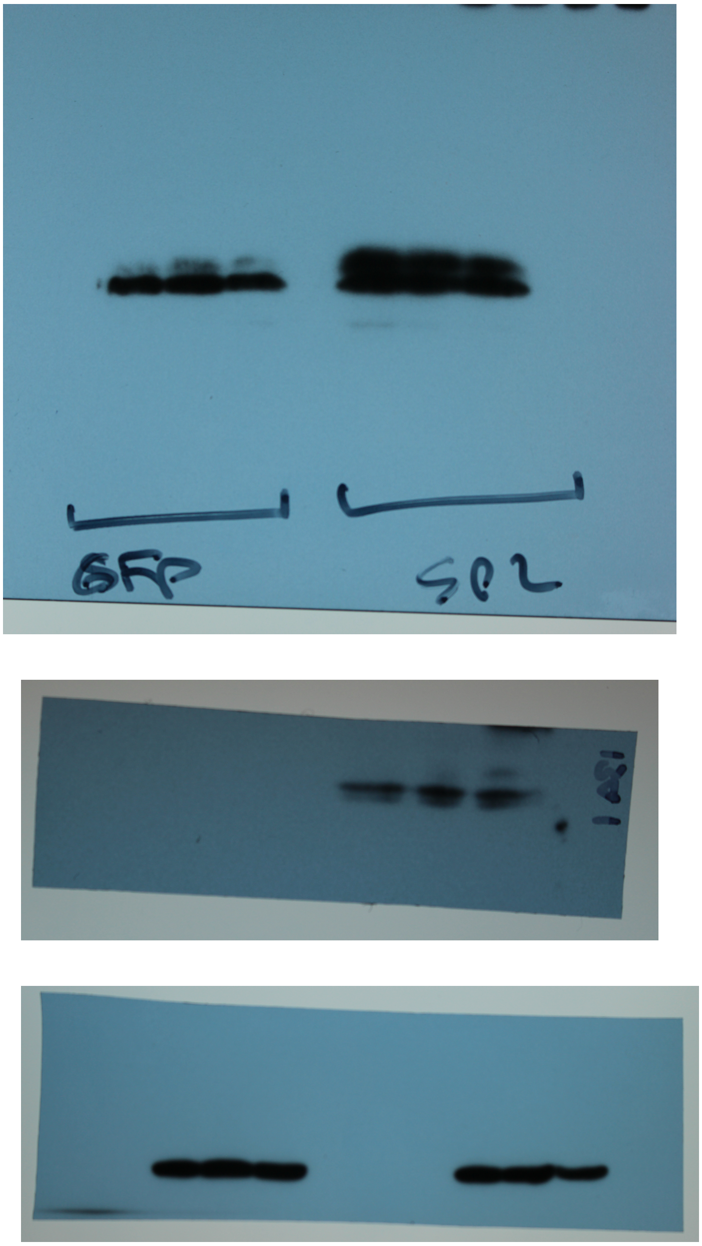

Supplement: Supplementary file 1 — Supplemental Figures [file 41531_2023_618_MOESM1_ESM.pdf]
